# Functional Analysis of the Two *Brassica AP3* Genes Involved in Apetalous and Stamen Carpelloid Phenotypes

**DOI:** 10.1371/journal.pone.0020930

**Published:** 2011-06-30

**Authors:** Yanfeng Zhang, Xuefang Wang, Wenxue Zhang, Fei Yu, Jianhua Tian, Dianrong Li, Aiguang Guo

**Affiliations:** 1 State Key Laboratory of Crop Stress Biology in Arid Areas, College of Life Science, Northwest A&F University, Yangling, Shaanxi, China; 2 Hybrid Rapeseed Research Center of Shaanxi Province, Shaanxi, China; University of Oxford, United Kingdom

## Abstract

The *Arabidopsis* homeotic genes *APETALA3* (*AP3*) and *PISTILLATA* (*PI*) are B genes which encode MADS-box transcription factors and specify petal and stamen identities. In the current study, the stamen carpelloid (SC) mutants, HGMS and AMS, of *B. rapa* and *B. napus* were investigated and two types of *AP3* genes, *B.AP3.a* and *B.AP3.b*, were functional characterized. *B.AP3.a* and *B.AP3.b* share high similarity in amino acid sequences except for 8 residues difference located at the C-terminus. Loss of this 8 residues in *B.AP3.b* led to the change of *PI*-derived motifs. Meanwhile, *B.AP3.a* specified petal and stamen development, whereas *B.AP3.b* only specified stamen development. In *B. rapa*, the mutations of both genes generated the SC mutant HGMS. In *B. napus* that contained two *B.AP3.a* and two *B.AP3.b*, loss of the two *B.AP3.a* functions was the key reason for the apetalous mutation, however, the loss-of-function in all four *AP3* was related to the SC mutant AMS. We inferred that the 8 residues or the *PI*-derived motif in *AP3* gene probably relates to petal formation.

## Introduction

The origin and evolution of petals in angiosperms remain elusive [1], [2]. Most flowers contain four types of floral organs, the sepal, petal, stamen and carpel, which are arranged in four concentric whorls. The classic ABC model proposes that three classes of floral homeotic genes coordinate with each other to specify the four floral organs: class A genes specify sepals, A+B genes together specify petals, B+C genes combine to specify stamens, and C genes alone specify carpels [3]–[7]. *APETALA3* (*AP3*) and *PISTILLATA* (*PI*), both MADS-box transcription factors, are class B genes in *Arabidopsis thaliana* and are involved in conferring petal and stamen identities. Mutations in these two genes exhibit similar homeotic conversions of petals to sepals and stamens to carpels [8]–[10].

Phylogenetic analyses suggest that the ABC class genes have undergone multiple duplication events and functional divergence during their evolution [11]–[15].The paralogous lineages *AP3* and *PI* arose from an ancestral class B gene duplication event before the origin of the angiosperms [16]–[19]. Subsequently, a major gene duplication event in the *AP3* lineage gave rise to the paralogous lineages *TM6* (tomato MADS box gene 6) and *euAP3* and coincided with the base of the higher eudicot radiation [20]–[22]. A number of higher eudicot species, such as tomato and petunia, contain both *euAP3* and *TM6* genes [21]–[23]. Interestingly, *Arabidopsis* and *Antirrhinum*, the two well-known model plants for studying flower development, contain only *euAP3* genes [24], [25]. In tomato, loss of *TAP3* (of the *euAP3* lineage) function results in the conversions of petals to sepals and stamens to carpels, but loss of *TM6* function only causes the homeotic transition of stamens to carpels and has little effect on perianth development [20]. In petunia, either *PhDEF* (*euAP3* lineage) or *PhTM6* (*Petunia hybrida TM6*) specifies stamen identity, but *PhDEF* has a redundant function of specifying petal development [25]–[26]. In the higher eudicots, similar to tomato and petunia, the *TM6* gene appears to specify stamen identity in the same way as the *paleoAP3* gene does, and the *euAP3* genes appear to specify stamen and petal identities [1]. In addition, the evolutionary origin of the higher eudicot petals coincides with a *TM6/euAP3* duplication event and the appearance of the *euAP3* genes [17], [27], [28]. This leads to the hypothesis that the *euAP3* genes have acquired a petal-specific function, compared with *paleoAP3* genes.

The *TM6* and *euAP3* lineages possess a distinct feature in their C-termini [24], [29]. Like paleo*AP3* lineages, the *TM6* lineage also contains a *paleoAP3* motif, which is present in *AP3* proteins throughout the lower eudicots, magnoliid dicots, monocots and basal angiosperms. In contrast, the *euAP3* lineage contains a *euAP3* motif, which is exclusively found in *AP3* proteins isolated from the higher eudicots and is most likely evolved from a translational frame shift mutation [29], [30]. A chimeric *euAP3* gene, which contains the *paleoAP3* C-terminal sequence instead of that of *euAP3* (including a *euAP3* motif and a *PI*-derived motif, which is defined as a region bearing similarity with the conserved *PI*-motif in the *PI* lineage), can partially rescue stamen development but is not sufficient to restore petal identity in the *ap3-3* mutant of *Arabidopsis* [24]. This suggests that the C-termini of *euAP3* proteins, particularly the *euAP3* motif and/or *PI*-derived motif, probably play a role in the capacity of eu*AP3* to specify petal development. However, two recent results also show that the C-terminal motif of *euAP3* is dispensable for its function in floral organ identity [31], [32]. So far, the exact mechanism behind the newly acquired role of *euAP3* in petal development is still uncertain.

When B class genes are lost, stamens are transformed into carpels and mutant plants exhibit a stable and complete male sterility phenotype. We are, therefore, interested in utilizing these male sterile mutant lines in our *Brassica* family hybrid breeding efforts. More recently, we isolated SC mutants from diploid *Brassica rapa* and allotetraploid *Brassica napus*, and bred a homeotic genic male sterile line (HMGS) of *B. rapa* and an apetalous male sterile line (AMS) of *B. napus* [33], [34]. Each of the HGMS and AMS lines displays a stably 1∶1 (fertile versus SC sterile plants) segregating line. In HGMS, the SC sterile plants exhibited transformations of petals into sepals and stamens into carpels, whereas, in AMS the SC plants showed petal-less and stamen-to-carpel phenotypes.

With these unique genetic resources in hand, we are in a good position to explore the mechanism of floral organ formation in *Brassica*, especially petal and stamen development. In the current report, a series of studies were performed, including genetic characterization, expression and sequence analysis of B class genes (*AP3* and *PI*) in HGMS and AMS lines as well as their hybrids. We show that two highly similar *AP3* genes (*B.AP3.a* and *B.AP3.b*) specified petal and stamen development in *Brassica*. Homeotic mutants of HGMS in *B. rapa* and AMS in *B. napus* were caused by loss of the two *AP3* functions, and the 24-bp sequence difference between them probably determined whether they specified petal formation.

## Results

### Origin and floral identities of SC mutants of *Brassica rapa* and *Brassica napus*

During our breeding experiments with the *B. rapa* variety “Wuyueman”, we recovered a natural SC mutant in a large selfing population. To maintain this mutant, we identified two kinds of maintainers in the same population as the SC mutant. The two maintainer plants pollinated the SC plant, and the resulting F_1_ generations both gave rise to fertile and SC sterile plants in a 1∶1 ratio. Each of the two lines was subsequently sustained by inter-sibling crossing at every generation, and the SC plants could be maintained at 50% at every generation (Table S1). The two 1∶1 segregated lines were named HGMS and HGMS2. The SC sterile plants in HGMS and HGMS2 had an identical phenotype and named HGMSa, whereas the two fertile maintainer plants were somewhat different and named HGMSb and HGMSb2, respectively.

The HGMSa mutation was also crossed with the fast-flowering *B. rapa* variety “Siyueman”, and we once again recovered anther different 1∶1 segregating line HGMSII from their F_2_ generation in the same way as the HGMS line described above. The fertile and SC sterile plants in HGMSII were named HGMSIIb and HGMSIIa respectively. The differences between HGMSII and HGMS could be largely attributed to their different genetic backgrounds.

**Figure 1:**
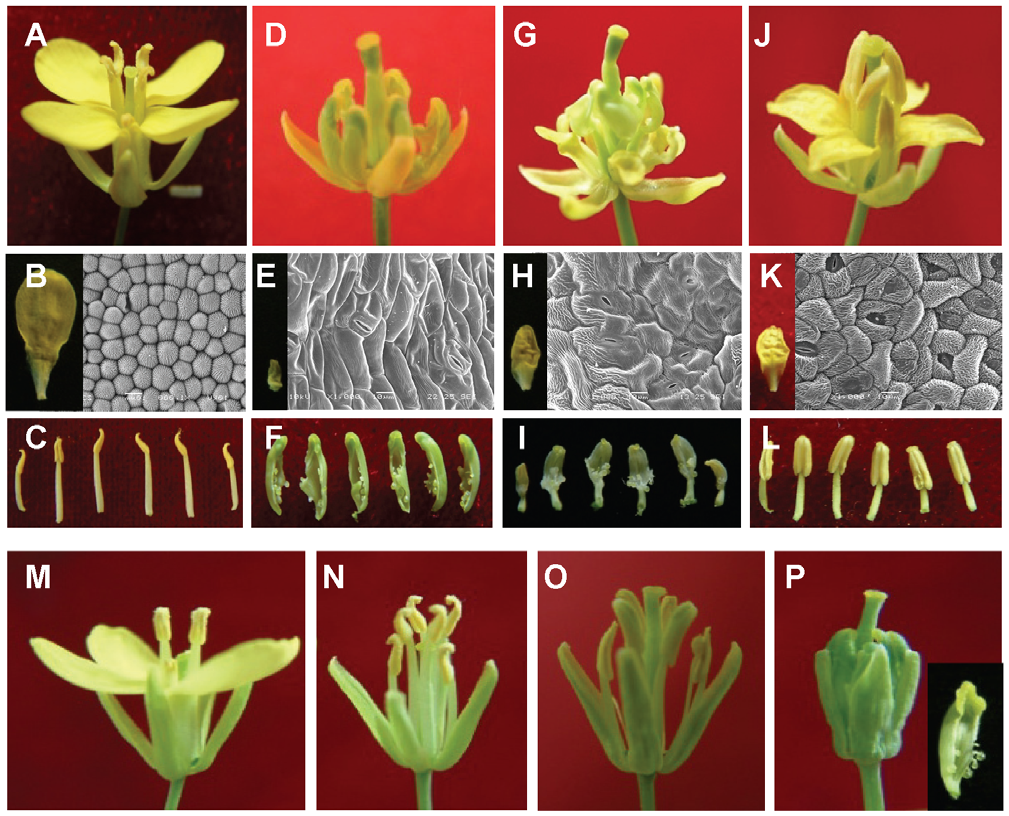
Flowers of wild-type and mutant *B. rapa* and *B. napus*. (A–L) *B. rapa* subsp. *chinensis* L. A,D,G,J: flowers. B,E,H,K: petals and their SEM photos (abaxial). C,F,I,L): stamens or SC organs. A, B, C: wild-type *B. rapa* (WTc). D, E, F: SC sterile plants of HGMS (HGMSa). G, H, I: SC sterile plants of HGMSII (HGMSIIa). J, K, L: another maintainer plant of HGMSa (HGMSb2). (M–P) *B. napus* L. M: wild-type *B. napus* (WTn). N: apetalous mutant of *B. napus* (Apt). O: fertile plants of AMS (AMSb). P: SC sterile plants of AMS (AMSa) and a carpel structure (in the bottom right corner).

The flowers of fertile HGMSb and HGMSIIb plants were morphologically similar to those of WTc (Wild type of *B. rapa*, Figure 1A–C). In HGMSa plants, however, the petals were extremely small, with elongated epidermal cells and stomas, both characteristics of the sepals rather than the petals (Figure 1D–E). In addition, HGMSa stamens were replaced by the carpels, with green ovule-like structures inside (Figure 1F). Thus, HGMSa flowers displayed a typical homeotic conversion of petals to sepals and stamens to carpels.

In HGMSIIa flowers, the petals were smaller than those of WTc and similar in size to the sepals, and they had irregularly shaped but elongated epidermal cells and stomas (Figure 1G–H), once again indicating a conversion of petals to sepals. The stamens also underwent a carpel-like transition. Interestingly, the transition was seemingly not as dramatic as that of HGMSa. The upper half of the carpel-like structure resembled the carpel with ovules, but at the base of the carpel-like structure were filament-like tissues (Figure 1I). Taken together, the phenotypes of HGMSIIa flowers display an incomplete transition of petals to sepals and stamens to carpels.

In the maintainer plant HGMSb2 (Figure 1J), the petals were sepal-sized and had malformed epidermal cells and stomas (Figure 1K), suggesting a transformation of petals into sepals. On the other hand, HGMSb2 stamens developed almost normally and produced pollen (Figure 1L).

During another breeding experiment in *B. napus*, we identified a petal-less mutant and bred a stably inherited apetalous line, Apt. In a large selfing population of Apt, we also recovered a natural SC mutant. By identifying a maintainer plant, we also established a 1∶1 segregating line, AMS (Table S1). The fertile and SC sterile plants in this line were named AMSb and AMSa, respectively. Besides the apparent petal-less phenotype, the flowers of the Apt line were otherwise identical to those of the wild-type WTn (Figure 1M–N). Reflecting the genetic background of Apt, the flowers of both AMSb and AMSa did not develop petals (Figure 1O–P). In addition to the petal-less phenotype, the stamens of AMSa were transformed into carpels with apparent ovule-like structures attached, whereas the stamens of AMSb developed almost normally (Figure 1P). The phenotype of AMSa suggested a homeotic conversion of stamens to carpels.

### Genetic characterization of SC mutants of *B. rapa* and *B. napus*

To determine the inheritance modes of our mutants, we carried out crosses between the mutants and different wild-type varieties of *B. rapa* and *B. napus*. In the case of *B. rapa*, the wild-type varieties Aj, Lx, ez1 and ey5 were crossed with HGMSa, and their F_1_ generations showed normal flowers, and SC sterile plants segregated in the F_2_ generations. The F_2_ segregation ratios of fertile and SC sterile plants in the four crosses were 13.4∶1, 13.5∶1 and 16.8∶1 and 14.9∶1, in accordance with a ratio of 15∶1 (Table 1). When F_1_ plants were backcrossed with sterile HGMSa plants, the segregation ratios of the four BC_1_ populations were 3.4∶1, 3.3∶1, 3.7∶1, and 3.0∶1, in agreement with a ratio of 3∶1. These results indicate that the SC phenotype of the HGMSa mutant was probably controlled by two pairs of recessive genes.

**Table 1:** Segregations of fertile and SC sterile plants in BC_1_ and F_2_ generations of HGMS hybrids and AMS hybrids.

Female parent	Male parent	BC_1_					F_2_				
		Fertile plant	Sterile plant	Fertile/sterile	Expected value	χ^2^	Fertile plant	Sterile plant	Fertile/sterile	Expected value	χ^2^
HGMS	Aj	518	154	3.4∶1	3∶1	1.45	389	29	13.4∶1	15∶1	0.23
	Lx	441	120	3.3∶1	3∶1	0.75	269	20	13.5∶1	15∶1	0.122
	ez1	129	35	3.7∶1	3∶1	0.98	252	15	16.8∶1	15∶1	0.09
	ey5	30	10	3.0∶1	3∶1	0.03	134	9	14.9∶1	15∶1	0.023
AMS	S3B	145	12	12.1∶1	15∶1	0.309	2532	10	253.2∶1	255∶1	0.019
	S4B	488	25	19.5∶1	15∶1	1.433	1455	7	207.9∶1	255∶1	0.109
	K407	369	27	13.7∶1	15∶1	0.132	1936	8	242∶1	255∶1	0.001

Note: χ^2^_0.05,1_ = 3.84; sterile plant means the SC sterile plant.

In the case of AMSa, when the wild-type *B. napus* varieties S3B, S4B and K407 were crossed with AMSa, their F_1_ generations showed normal flowers, and SC sterile plants segregated in the F_2_ generation. The F_2_ segregation ratios of fertile and SC sterile plants of the three crosses were 253.2∶1, 207.9∶1 and 242∶1, supporting a ratio of 255∶1 (Table 1). When F_1_ plants were backcrossed with AMSa plants, the segregation ratios of the BC_1_ populations in the three crossings were 12.1∶1, 19.5∶1 and 13.7∶1, fitting a ratio of 15∶1. These results indicate that the SC phenotype of the AMSa mutant was likely controlled by four pairs of recessive genes.

### The *PI* gene is expressed normally in SC mutants of *B. rapa* and *B. napus*

Although the genetic mechanism behind the SC phenotypes of HGMSa and AMSa were different, both of them had the same SC phenotypes. According to the classic ABC model, class B genes (*AP3* and *PI*) specify petal and stamen development. Mutations of *AP3* and/or *PI* genes in *Arabidopsis* exhibit similar homeotic transformations of petals to sepals and stamens to carpels [3], [10]. Surprisingly, our mutants also displayed similar homeotic transformations as those of *AP3* and/or *PI* mutants, so we examined the expression pattern of *AP3* and *PI* genes in these mutants of *B. rapa* and *B. napus*.

To investigate *PI* expression, we carried out semi-quantitative RT-PCR amplifications using cDNAs from HGMSa, AMSa and wild-type plants of *B. rapa* and *B. napus* as templates. With *PI*-specific primers, all samples gave an apparently identical RT-PCR amplification product. Subsequent sequencing of the RT-PCR products revealed 100% identity of *PI* sequences among the mutants and the wild-type plants. These data indicate that the *PI* expression did not change in SC mutants of *B rapa* and *B. napus* (results not shown).

### Expression and distribution of two types of *AP3* genes in mutants of *B. rapa*

**Figure 2:**
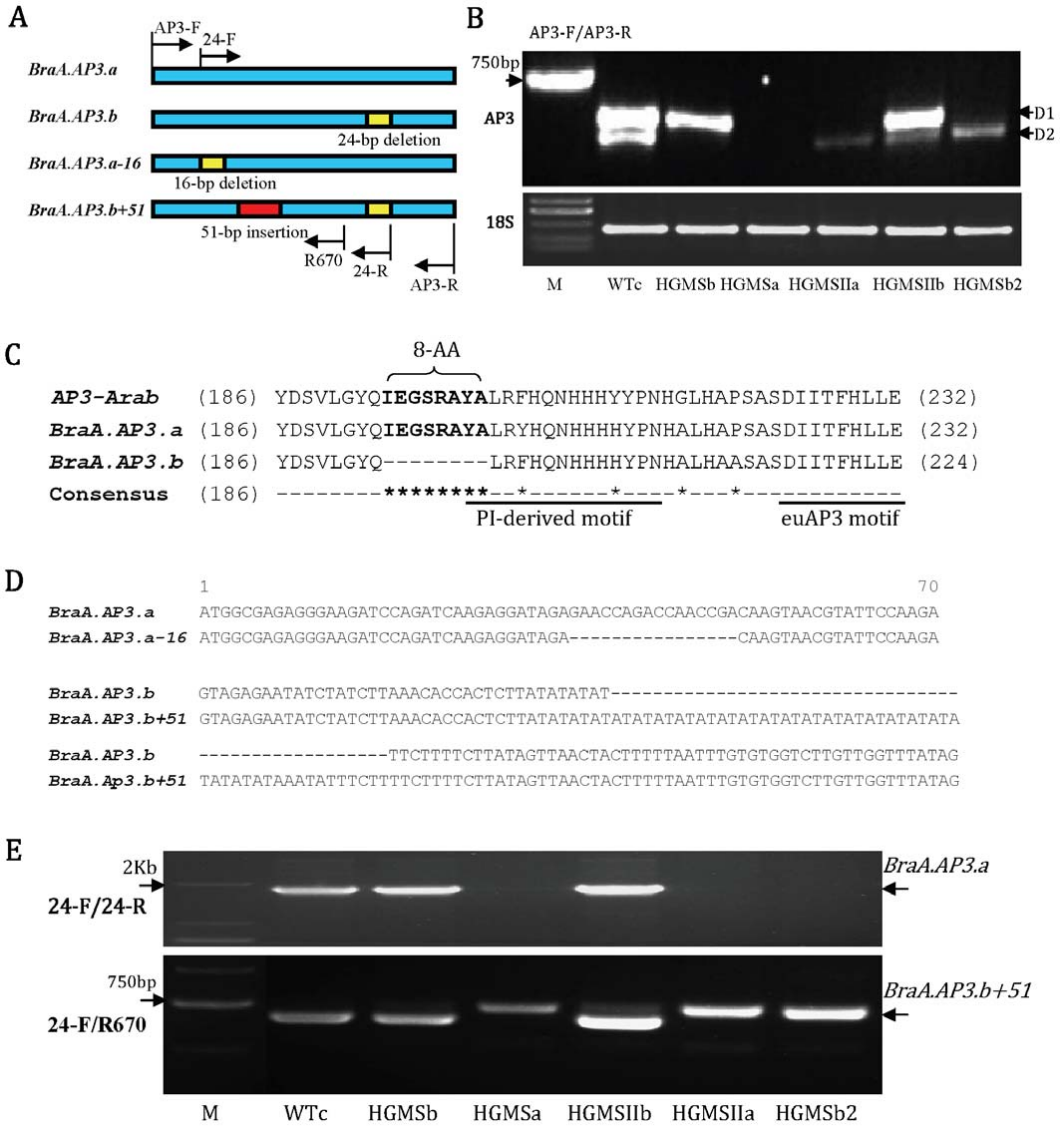
Expression and alignment of the two *AP3* genes of wild-type and mutant *B. rapa.* (A) Structure schematic of the *AP3* genes with the primer position; (B) Expression of the two *AP3* genes. D1 and D2 correspond to the *BraA.AP3.a* and *BraA.AP3.b* genes, respectively; (C) Partial amino acid alignment of *BraA.AP3.a* and *BraA.AP3.b* as well as *AP3* from Arabidopsis (*AP3-Arab*); (D) Alignment of the two *AP3* genome DNA (partial sequences) of wild-type and mutant *B. rapa*. In mutant *B. rapa*, the *BraA.AP3*.a has a 16-bp deletion in its first exon, named *BraA.AP3.a-16*, and the *BraA.AP3.b* has a 51-bp insertion, which contains a 40-bp AT repeat sequence, in its first intron, named *BraA.AP3.b+51*; (E) DNA amplification of wild-type and mutant *B. rapa* using 24-F/24-R primers (up) and 24-F/R670 primers (down), respectively.

To examine *AP3* expression, semi-quantitative RT-PCR were also performed using the *AP3*-specific primers AP3-F and AP3-R. We detected two major PCR products from wild-type *B. rapa* (WTc) and named the longer fragment as D1 and the shorter fragment as D2 (Figure 2A). In contrast to WTc, the maintainer plant HGMSb only contained one major D1 band. The maintainer plant HGMSIIb had a strong D1 band but also had a very weak D2. The differences among HGMSa, HGMSIIa, HGMSb2 and WTc were much more dramatic. We did not detect any *AP3* RT-PCR products in HGMSa, but only a very low level of D2 in HGMSIIa was detected. The maintainer plant HGMSb2 contained only a D2 band, and its expression was higher than in HGMSIIa but lower than in WTc. The expression of cytosolic 18S rRNA gene was used as a control, and its expression was comparable in all samples (Figure 2A).

Sequence analysis of all RT-PCR samples showed that D1 bands from WTc, HGMSb and HGMSIIb were all identical, with a length of 699 bp. D2 bands from WTc and HGMSIIb and HGMSIIa and HGMSb2 were all identical, with a length of 675 bp.

Interestingly, the nucleotide sequences of D1 and D2 showed 91% identity. The differences between D1 and D2 included 38 single-nucleotide polymorphisms (SNP) (Figure S1), three of which caused amino acid substitutions, as well as a conspicuous 24-bp (8 amino acid residues) insertion/deletion near their C-termini (Figure 2B; Figure S1 and S2). Blasting the D1 and D2 sequences at the *B.rapa* genome database (http://brassicadb.org.brad/), the result revealed that the two sequences were same as two *AP3* genes Bra007067 and Bra014822 (Figure S1 and S2), which located at the A09 and A04 chromosomes of *B.rapa*, respectively. Meanwhile the D1 and/or D2 sequences had also been identified in *B. napus* (DQ372719, AY313940, DQ372720, AF124814 and AY313941), *B. rapa* (AY623003), *B. oleracea* (U67453 and U67455) and *B. juncea* (DQ060332). And we also confirmed the presence of D1 and D2 in the four species of *Brassica* by RT-PCR (data not shown). These indicate that the D1 and D2 RT-PCR products represented two homologous *AP3* genes in *B. rapa*. We thus named the two genes *BraA.AP3.a* and *BraA.AP3.b* according to the standardized gene nomenclature for the *Brassica* genus,which built by Ostergaard and King (2008) [35].

Further amplification of genomic DNAs from HGMSa and WTc showed that *BraA.AP3.a* of HGMSa had a 16-bp deletion in the first exon from bp 36 to 51 (*BraA.AP3.a-16*; Figure 2C and Figure S3), and *BraA.AP3.b* of HGMSa had a 51-bp insertion, which contained a 40-bp AT repeat sequence, in the first intron (Figure 2C and Figure S3). With the designed forward primer 24-F at the 16-bp deletion region of *BraA.AP3.a-16* and reverse primer 24-R with the special 24-bp sequence of *BraA.AP3.a* to amplify genomic DNAs of all the materials (Figure 2D), WTc, and HGMSb and HGMSIIb all showed one clear *BraA.AP3.a* band. However, no bands were detected in HGMSa, HGMSIIa and HGMSb2, indicating that the *BraA.AP3.a* genes in them underwent a 16-bp deletion.

We designed another reverse primer, R670, against a region after the first intron of *BraA.AP3.a*, and used 24-F and R670 primers to amplify genomic DNAs of all the materials again (Figure 2D). WTc showed one band (containing *BraA.AP3.a* and *BraA.AP3.b*), whereas HGMSa, HGMSIIa and HGMSb2 lost this band, but each had an additional *BraA.AP3.b+51* band, which was slightly larger than that of WTc, indicating that the *BraA.AP3.b* gene in them underwent a 51-bp insertion.

Take together, the above results show that mutations in *BraA.AP3.a* and *BraA.AP3.b* were related to SC mutants of *B. rapa*.

### Expression and distribution of four *AP3* genes in mutants of *B. napus*

**Figure 3:**
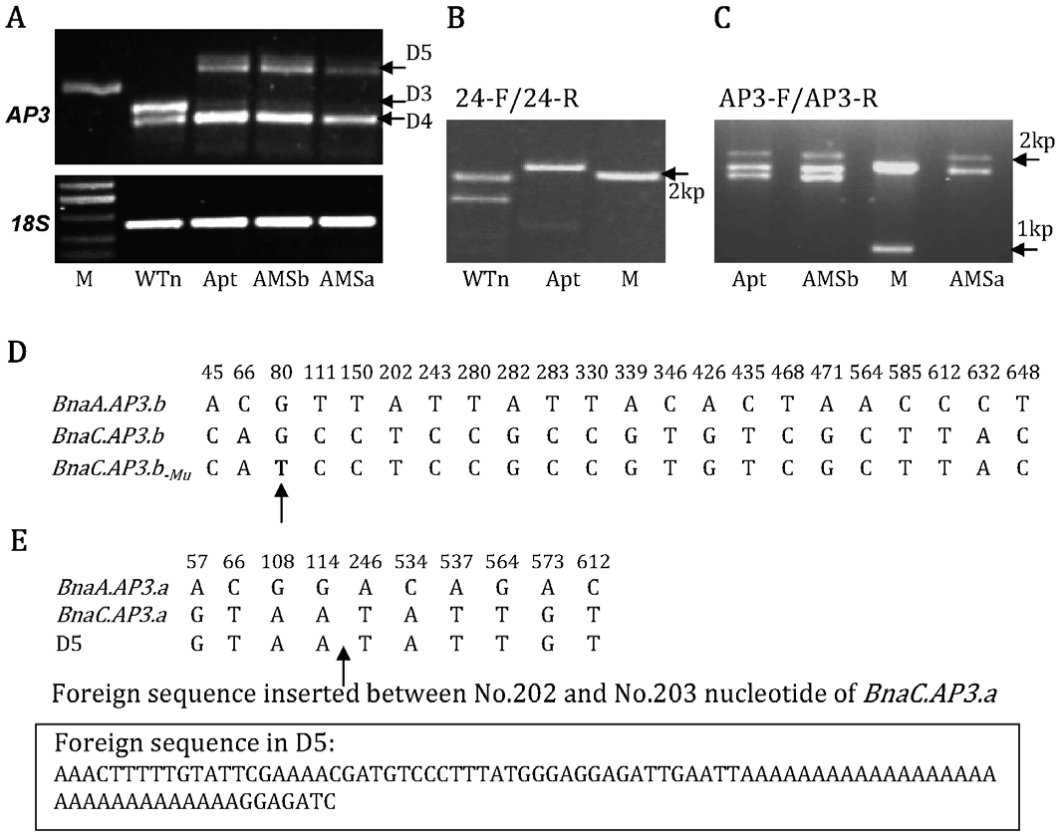
Expression and alignment of the *AP3* genes in wild-type and mutant *B. napus*. (A) Expression of *AP3* genes in wild-type and mutants of *B. napus*; (B) DNA amplification of WTn and Apt with the 24-bp sequence specific primers 24-F and 24-R; (C) DNA amplification of Apt, AMSb and AMSa with AP3-F and AP3-R primers; (D) Nucleic acid alignment of *BnaA.AP3.b* and *BnaC.AP3.b* of *B. napus* (only the SNPs are shown). *BnaC.AP3.b_-Mu_* is the *BnaC.AP3.b* gene with a G–T single-nucleotide mutation (shown by an arrow); (E) Nucleic acid alignment of *BnaA.AP3.a* and *BnaC.AP3.a* of *B. napus* (only the SNPs are shown); D5 is the *BnaC.AP3.a* gene with an 86-bp foreign insertion in its second exon (shown by an arrow).

The aberrant expression of *BraA.AP3.a* and *BraA.AP3.b* in our *B. rapa* mutants prompted us to look into the expression of *AP3* homologous genes in our *B. napus* mutants. *AP3* homologues in *B. napus* were amplified by semi-quantitative RT-PCR using the *AP3*-specific primers AP3-F and AP3-R. We again detected two bright PCR bands in the wild-type *B. napus* (WTn, Figure 3A), which were named D3 and D4, respectively. In contrast, Apt, AMSb and AMSa plants lacked D3 but contained D4 and one additional band of approximately 800-bp, which we named D5.

To further investigate the natures of the D3, D4 and D5 bands, we sequenced them and found that the D3 band of WTn was 699-bp, and the D4 bands of WTn, Apt, AMSb and AMSa were all 675-bp long, whereas the D5 bands of Apt, AMSb and AMSa were 785-bp.

Further sequence analysis revealed that D4 of WTn contained two kinds of highly homologous *AP3* genes (96.7% identity and 21 single-nucleotide polymorphisms, two of which changed amino acids; Figure 3D and Figures S4, S5). Of the two *AP3* genes, one was identical to the *BraA.AP3.b* gene of *B. rapa* as well as the *AP3* sequences of *B. rapa* (AY60003) and *B. napus* (DQ372720); the other shared 99.4% identity with *AP3* genes in *B. oleracea* (Bou67455) and *B. napus* (AY313941), and contained only two SNP (one amino acid substitution; data not shown). These results indicate that WTn of *B. napus* contains two types of *AP3* genes with the 24-bp deletion identity: one is identical to *BraA.AP3.b* of *B. rapa*, named *BnaA.AP3.b*; whereas the other, which we named *BnaC.AP3.b*, is highly homologous to that of *B. oleracea* (Figure S4).

Similarly, we also found that D3 sequences of WTn also contained two types of *AP3* genes (97.4% identical and 10 single-nucleotide polymorphisms, and no amino acid change; Figure 3E and Figures S5, S6). One was the same as the *BraA.AP3.a* gene of *B. rapa*, the other was the same as the *AP3* gene of *B. oleracea* (Bou67453). After using 24-F and 24-R primers, which specifically amplified *AP3* genes with the 24-bp special sequence, to amplify genomic DNA from WTn, two clear PCR bands were obtained (Figure 3B), confirming that WTn of *B. napus* contains two *AP3* genes: one is identical to *BraA.AP3.a* of *B. rapa*, and the other, which we named *BnaC.AP3.a*, is the same as that of *B. oleracea*. Taken together, we have identified four *AP3*-like genes from *B. napus*: *BnaA.AP3.b*, *BnaC.AP3.b*, *BnaA.AP3.a* and *BnaC.AP3.a* (Figure S4 and S5).

Sequencing of the D5 band from Apt plants showed that the D5 sequence was exactly the same as *BnaC.AP3.a* except for an 86-bp insertion in its second exon (Figure 3E and S6). Using the 24-bp-specific primers 24-F and 24-R, only one clear band could be amplified from Apt genomic DNA, which was slightly larger than the *BnaC.AP3.a* band of WTn, and sequencing confirmed that this band corresponded to the D5 band of RT-PCR (Figure 3B). These findings suggest that Apt had at least lost the *BnaA.AP3.a* copy of the *AP3* gene and that the *BnaC.AP3.a* copy of *AP3* contained an 86-bp insertion. Subsequently, sequencing of the Apt D4 band showed that Apt contained both *BnaA.AP3.b* and *BnaC.AP3.b*. *BnaA.AP3.b* of Apt was the same as that of WTn. However, when compared with WTn, *BnaC.AP3.b* of Apt contained a single-nucleotide mutation in which the 80th nucleotide transformed from G into T and the corresponding 27th amino acid from glycine into valine, and this mutant form of Apt *BnaC.AP3.b* was named *BnaC.AP3.b_-Mu_* (Figure 3D and S4). Taken together, the loss of the *BnaA.AP3.a* gene, along with the mutations of *BnaC.AP3.a* and *BnaC.AP3.b*, was correlated with the apetalous identity of Apt. We did not detect a mutation or expression change of *BnaA.AP3.b*, suggesting that this gene was still functional and probably responsible for the normal development of Apt stamens.

The possible losses of the three *AP3*-like genes in Apt raised the question about the fate of the fourth *AP3* gene *BnaA.AP3.b* in the SC mutant AMSa. To address this question, we carried out PCR amplification of Apt, AMSb and AMSa genomic DNA templates with AP3-F and AP3-R primers. We observed three bands in Apt and AMSb. In AMSa, we detected the top two bands, but not the smallest fragment (Figure 3C). Sequencing analysis showed that the three bands found in Apt and AMSb corresponded to D5, *BnaC.AP3.b_-Mu_* and *BnaA.AP3.b*, whereas the two bands in AMSa corresponded to D5 and *BnaC.AP3.b_-Mu_*, with the *BnaA.AP3.b* band absent in AMSa (Figure 3C). The failure to amplify *BnaA.AP3.b* from AMSa suggests that it may have been lost. As we indicated earlier, the Apt background was associated with the disturbance of three *AP3* genes: *BnaA.AP3.a*, *BnaC.AP3.a* and *BnaC.AP3.b*. The loss of the fourth *AP3* gene, *BnaA.AP3.b*, and the phenotype of AMSa plants suggest that the disruption of all four *AP3* genes is correlated with the SC identity of AMSa.

### Two types of *AP3* genes controlling stamen and/or petal development

The amino acid sequences of *BraA.AP3.a* and *BnaA.AP3.a* and *BnaC.AP3.a* were identical, and those of *BraA.AP3.b* and *BnaA.AP3.b* and *BnaC.AP3.b* were also the same except for three amino acid differences (Figure S5). For simplicity, *BraA.AP3.a* and *BnaA.AP3.a* and *BnaC.AP3.a* were combined as *B.AP3.a*, whereas *BraA.AP3.b* and *BnaA.AP3.b* and *BnaC.AP3.b* were combined as *B.AP3.b*. The SC mutants of *B. rapa* and *B. napus* were all related to abnormal *B.AP3.a* and *B.AP3.b* gene expression. To further investigate the functions of the two genes, SC mutants of *B. rapa* and *B. napus* were reciprocally crossed.

**Figure 4:**
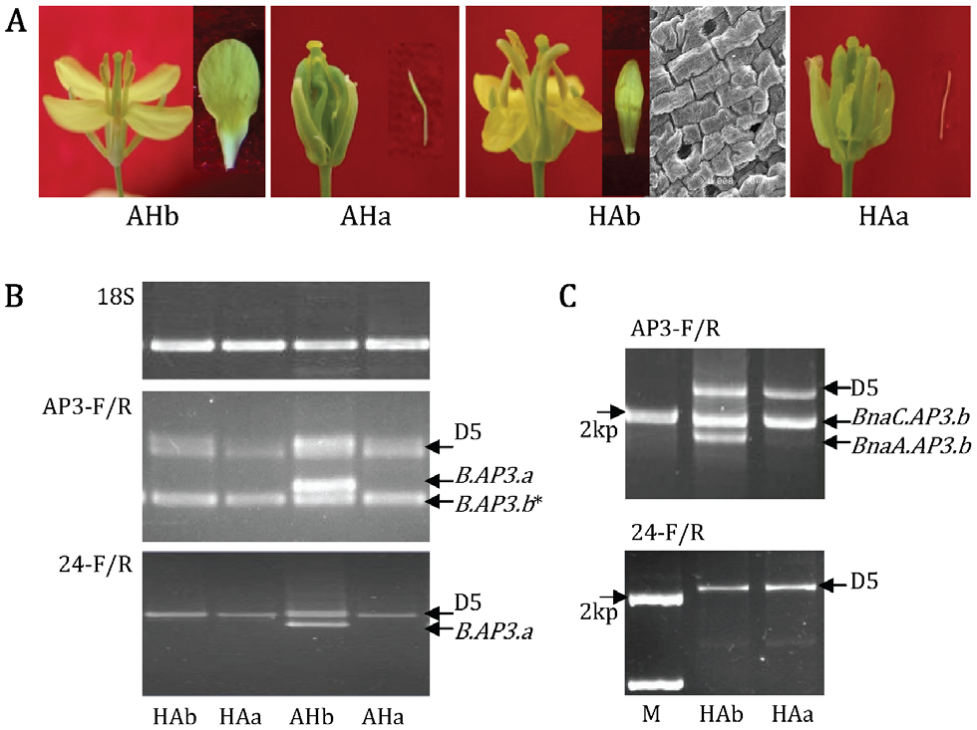
Flowers and *AP3* gene expression of F_1_ hybrids between AMS and HGMS. (A) Flowers and petals of F_1_ hybrids between AMS and HGMS. Fertile and SC sterile plants from AMSa×HGMSb were named AHb and AHa, respectively, whereas those from HGMSa×AMSb were named HAb and HAa, respectively; (B) Expression of *AP3* genes in HAb, HAa, AHb and AHa (primers AP3-F/AP3-R and 24-F/24-R for amplification). 18S rRNA served as the control. The AHb plants carried the *B.AP3.a* gene (showed by an arrow), which came from male parent HGMSb; their petals and stamens developed normally. *B.AP3.b band includes *BnaC.AP3.b_-Mu_* and/or *BnaA.AP3.b* sequences; (C) DNA amplification of HAb and HAa with AP3-F/R (up) and 24-F/R (down) primers. The HAb plants contained the *BnaA.AP3.b* gene (showed by an arrow), which came from the male parent AMSb, their stamens developed normally, but petals transformed into sepals.

In the F_1_ generation of AMSa×HGMSb, HGMSa×AMSb, AMSa×HGMSIIb and HGMSIIa×AMSb, fertile and SC plants segregated at a ratio of 1∶1 (F_1_ plants designations are indicated in Table 2 and Figure 4A). This segregation ratio indicated that the gene loci that caused SC mutations of AMSa and HGMSa and HGMSIIa were the same and were probably situated on the A-genome chromosome.

**Table 2:** Fertile plants and SC sterile plants of F_1_ hybrids of AMS with HGMS and HGMSII.

Combination	Fertile plant		Sterile plant		Fertile/sterile	Expected value	χ^2^
	name	number	name	number			
AMSa×HGMSb	AHb	36	AHa	35	1.029∶1	1∶1	0.01
HGMSa×AMSb	HAb	11	HAa	19	0.579∶1	1∶1	2.13
AMSa×HGMSIIb	AHIIb	49	AHIIa	56	0.875∶1	1∶1	0.47
HGMSIIa×AMSb	HAIIb	20	HAIIa	37	0.541∶1	1∶1	5.07

Note: Sterile plants: SC sterile plants. Fertile plant and sterile plant were HAb and HAa in HGMSa×AMSb, AHb and AHa in AMSa×HGMSb, AHIIb and AHIIa in AMSa×HGMSIIb, and HAIIb and HAIIa in HGMSIIa×AMSb.

Due to the abolished expression of *B.AP3.a* and *B.AP3.b*, HGMSa exhibited homeotic transformations of petal into sepal and stamen into carpel. In contrast, HGMSb expressed just one *B.AP3.a* gene, and its petal and stamen developed normally. In AMSa×HGMSb, fertile plants AHb had a normal-expressing *B.AP3.a* gene, which came from male parent HGMSb (Figure 4B), and their petals and stamens developed normally in comparison with SC plants AHa (Figure 4A). Similarly, in AMSa×HGMSIIb, the fertile plants AHIIb also had a functional *B.AP3.a* gene from HGMSIIb (Figure S7), and thus their petals and stamens also developed normally. These results indicate that the *B.AP3.a* gene is sufficient to specify petal and stamen development.

The *B.AP3.b* gene was not expressed in AMSa, and this mutant line exhibited petal loss and SC identity. An AMSb line with a normal-expressing *B.AP3.b* gene showed an apetalous identity, and stamens developed normally. In HGMSa×AMSb, sterile AHa plants exhibited petal loss and SC identity, and fertile HAb plants showed a petal-to-sepal transition while stamens developed normally. Further, genomic DNA of HAb and HAa were amplified using AP3-F/R primers, and an extra band was obtained from the HAb plant (Figure 4C). Sequencing showed that the band was the genomic DNA of *BnaA.AP3.b*, which came from the male parent AMSb. Similarly, in HGMSIIa×AMSb, the fertile HAIIb plants with sepaloid petals and normally developed stamens also had a *BnaA.AP3.b* gene from AMSb (Figure S7). These data show that the only function of *B.AP3.b* is to specify stamen development and it has little effect on petal formation.

## Discussion

### Two types of *AP3* genes specify petal and stamen development of *Brassica*

*B. napus* (*AACC* genome) is an allotetraploid species that originated from a spontaneous hybridization between *B. rapa* (*AA* genome) and *B. oleracea* (*CC* genome) and possesses the complete diploid chromosome sets of both parental genomes [36], [37]. Our results reveal two kinds of *AP3* genes, *B.AP3.a* and *B.AP3.b*, specifying petal and stamen development in *B. rapa* and *B. oleracea*. It stands to reason that *B. napus* has four *AP3* genes: *BnaA.AP3.a* and *BnaA.AP3.b* from *B. rapa* and *BnaC.AP3.a* and *BnaC.AP3.b* from *B. oleracea*. Therefore, this is a new evidence regarding the origin of *B. napus*.

Our results indicate that *B.AP3.a* specifies petal and stamen development, in parallel with *AP3* function in *Arabidopsis* [3], and *B.AP3.b* only specifies stamen development, which concurs with the research results of Pylatuik (2003): when the AG::BnAP3 construct (*B.AP3.b* gene with an *agamous* promoter) was translated into *ap3*-1 mutants, it restored stamen development, but not petal development [38].

### Mutations of *B.AP3.a* and *B.AP3.b* genes causing the SC mutants of *B. rapa*

**Figure 5:**
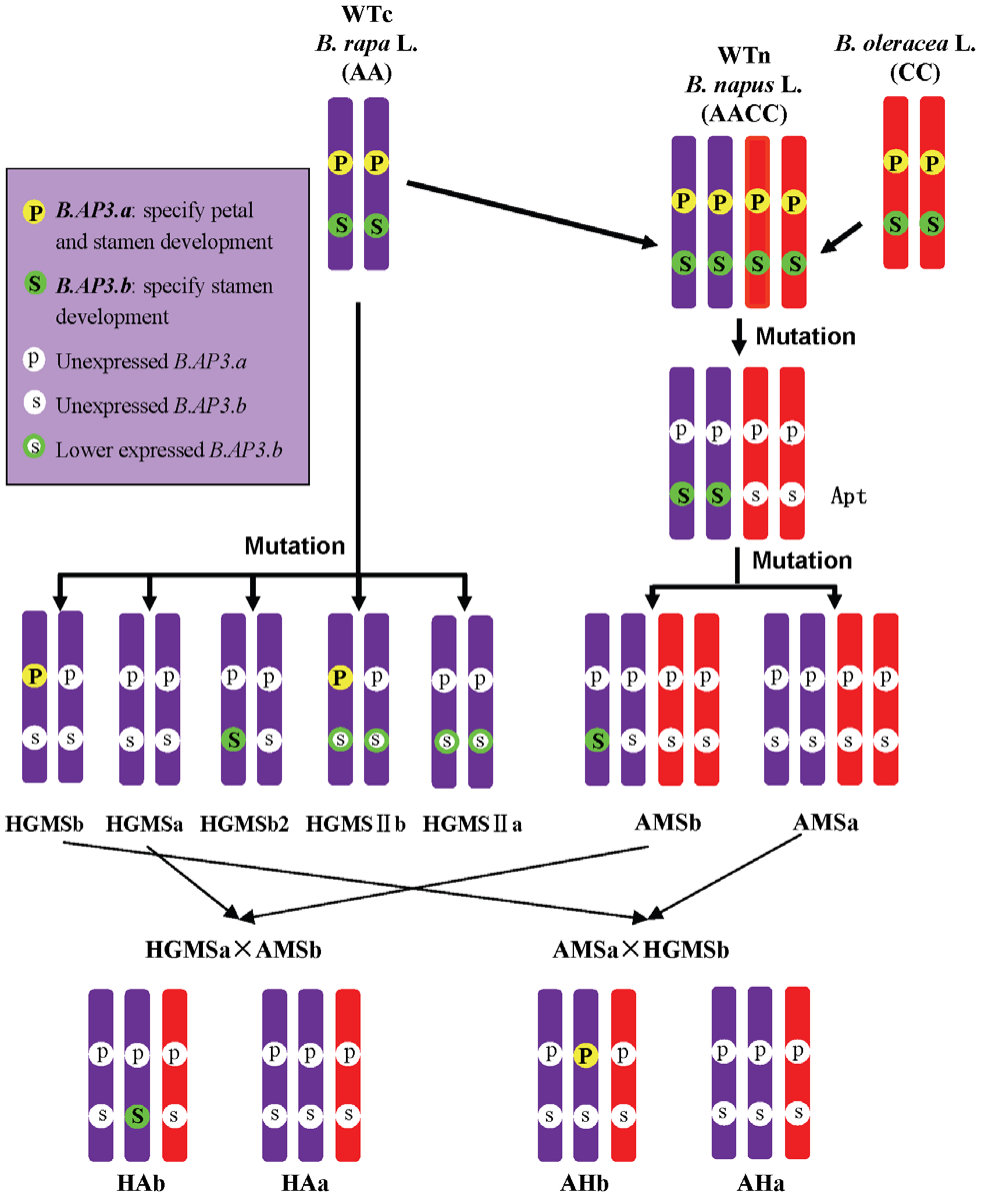
*AP3* genes distribution in wild-type and mutant *B. rapa* and *B. napus*.

With a 16-bp sequence deletion from the first exon of *BraA.AP3.a* and a 51-bp foreign sequence insertion in the first intron of *BraA.AP3.b*, the two *AP3* genes were totally unexpressed, leading to a homeotic transition in HGMSa from petals to sepals and stamens to carpels. This is consistent with the genetic result that SC identity in HGMSa is controlled by two pairs of recessive genes: *B.AP3.a* and *B.AP3.b*. HGMSb expressed only a single *B.AP3.a* gene, which specified petal and stamen formation, so its petals and stamens developed normally. Moreover, HGMSb2 expressed only a single *B.AP3.b* gene, which specified only stamen formation, so its stamens developed normally and petals transformed into sepals. If P and S stand for *B.AP3.a* and *B.AP3.b*, and p and s stand for the mutant genes of *B.AP3.a* and *B.AP3.b*, the genotypes of HGMSa, HGMSb and HGMSb2 are presumed to be ppss, Ppss and ppSs, respectively (Figure 5).

HGMSb2 had one *B.AP3.b* gene, thus possessing normally developed stamens, and HGMSIIa had one low-expressed *B.AP3.b* gene, thus exhibiting stamen to carpel transformation. This indicates that the expression level of *B.AP3.b* is related to stamen development. Where *B.AP3.b* expression was decreased, it lost its function so that SC mutants were created. Therefore, HGMSIIa is a petal sepaloid and SC mutant resulting from the non-expression of *B.AP3.a* and lower expression of *B.AP3.b*. Therefore, it is assumed that the genotypes of HGMSIIb and HGMSIIa were Ppss and ppss (Figure 5).

### Losing the two *B.AP3.a* functions is the key reason for the apetalous mutant Apt of *B. napus*

In Apt, *BnaA.AP3.a* was lost, the second exon of *BnaC.AP3.a* had an 86-bp foreign sequence, and surprisingly, the foreign sequence contained a 29-bp polA sequence. It is assumed that the foreign insertion probably led to a loss of *BnaC.AP3.a* function. Meanwhile, *BnaC.AP3.b* of Apt mutated at a single nucleotide, thus changing a single amino acid, which lay in an extremely conserved MADS-box domain. Thus, the mutation probably caused a loss of function of *BnaC.AP3.b*. These results show that losses of function of the three *AP3* genes correlated with the apetalous mutant Apt, and losing the two *B.AP3.a* gene functions, which specially specified petal development, was the key reason for the petal-loss identity of Apt. In addition, *BnaA.AP3.b* of Apt was identical to that of WTn, and this was a possible reason for normal stamen development in the Apt plant.

### Loss of four *AP3* functions causing the SC mutant AMSa of *B. napus*

AMSb and AMSa originated from Apt and carried loss-of-function mutations of *BnaA.AP3.a*, *BnaC.AP3.a* and *BnaC.AP3.b*. Compared with AMSb, AMSa once again lost *BnaA.AP3.b*. As a result, the four *AP3* genes of AMSa totally lost their functions. This was the primary reason for the SC mutant AMSa and was consistent with the genetic result that SC identity in AMSa was controlled by four pairs of recessive genes. These four pairs of genes of AMSa were the four *AP3* genes: two *B.AP3.a* and two *B.AP3.b*. It is presumed that the genotypes of AMSa, AMSb, Apt and WTn were ppppssss, ppppSsss, ppppSSss and PPPPSSSS, respectively (Figure 5).

### The 24-bp insertion or *PI*-derived motif probably plays a key role in petal formation

*PaleoAP3* and *euAP3*, although they share significant sequence similarity, are characterized by the *PI*-derived motif and *AP3* motif in the C- termini of their proteins. *PaleoAP3* lineage gene (including TM6) contains *paleoAP3* motif, whereas *euAP3* lineage gene has a *euAP3* motif instead of the *paleoAP3* motif. *PaleoAP3* specifies stamen development, but *euAP3* specifies petal and stamen development [1], [17], [24]. Does the *euAP3* motif contribute to petal development? *B.AP3.a* and *B.AP3.b* had the same *euAP3* motif (Figure 2B), and if the motif is related to petal formation, the two genes should certainly have the function of specifying petal development. However, the results of Pylatuik et al. (2003) and this study show that *B.AP3.b* probably does not specify petal formation [38]. Therefore, the *euAP3* motif in the context of *B.AP3.b* is insufficient for petal development.

Does the *PI*-derived motif contribute to petal development? *B.AP3.a* and *B.AP3.b* are highly homologous while having a notable difference in function. Obviously, their sequence differences may contribute to their functional divergence. Surprisingly, the 24-bp divergence gave rise to different *PI*-derived motifs between *B.AP3.a* and *B.AP3.b* (Figure 2B). Again analyzing the research results of Lamb & Irish (2003), we found that where the C-terminus (amino acids 200–232) of *AP3* is truncated, the 8-amino acid sequence was divided into two parts and damaged the *PI*-derived motif of *euAP3* [24]. This might have led the constructed *AP3_CPALEO_* to lose its function of regulating petal development. These data indicate that the 24-bp sequence or *PI*-derived motif probably relates to petal development.

Due to the 24-bp difference existing natively and exhibiting two statuses before and after *AP3* mutation, *B.AP3.a* may have originated from *B.AP3.b* with the 24-bp foreign insertion, and the insertion is probably related to the origin of the petal.

### ABC model of *Brassica* flower development is controlled by two types of *AP3* genes

**Figure 6:**
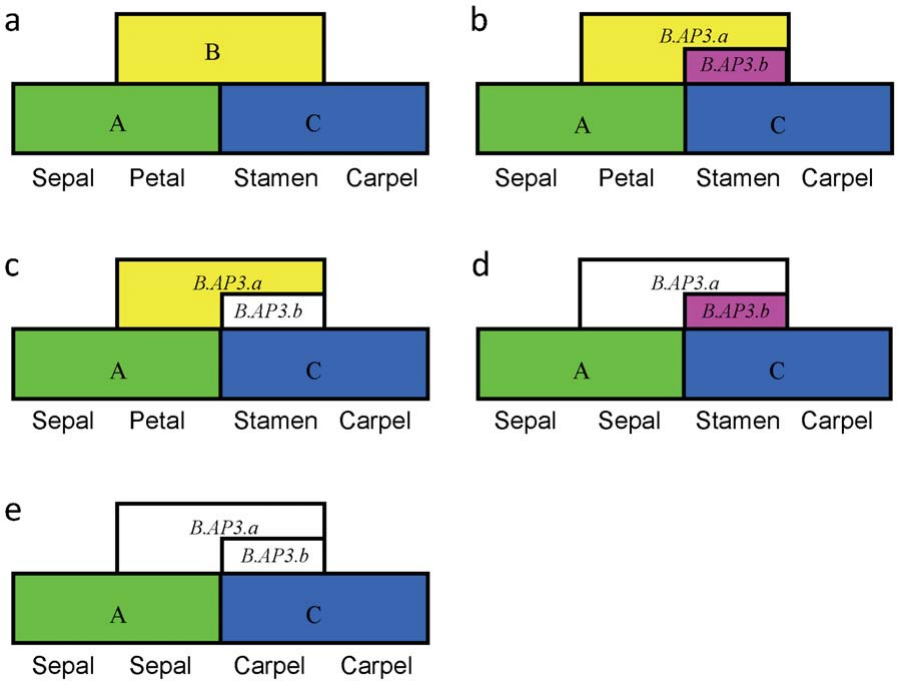
Floral ABC model of *Brassica* under the control of two types of *AP3* genes. (a) Classic ABC model of flower development [3]; (b) ABC model of *Brassica* flower development under the control of *B.AP3.a* and *B.AP3.b* genes. *B.AP3.a* specifies petal and stamen development, whereas *B.AP3.b* specifies only stamen development. When *B.AP3.b* is lost and only *B.AP3.a* is expressed, its petals and stamens also develop normally (c); when *B.AP3.a* is lost and only *B.AP3.b* is expressed, its stamens develop normally, but petals transform into sepals (d); when *B.AP3.a* and *B.AP3.b* are both lost, the petal-to-sepal and stamen-to-carpel homeotic transitions occur (e).

There are two types of *AP3* genes, *B.AP3.a* and *B.AP3.b*, specifying petal and stamen development in *Brassica*. The classic ABC model (Figure 6a) cannot perfectly explain floral development in *Brassica*. To make the model more suitable for *Brassica* plants, the two kinds of *AP3* genes should be considered. *B.AP3.a* specifies petal and stamen development, whereas *B.AP3.b* specifies only stamen development. Usually, the two genes express together to specify petal and stamen development in *Brassica* plants (Figure 6b). With *B.AP3.b* lost and only *B.AP3.a* expressed, normal petals and stamens develop (Figure 6c); with *B.AP3.a* lost and only *B.AP3.b* expressed, stamens develop normally, but its petals transform into sepals (Figure 6d); and with both *B.AP3.a* and *B.AP3.b* lost, homeotic conversions of petals to sepals and stamens to carpels occur (Figure 6e).

## Materials and Methods

### Plant materials

#### *B. rapa* materials

Wild-type (WTc) *B. rapa* subsp. *chinensis* and three types of SC sterile 1∶1 lines (HGMS, HGMS2 and HGMSII) of *B. rapa* were used.

#### *B. napus* materials

Wild-type (WTn) *B. napus*, apetalous mutant of *B. napus* (Apt) and apetalous SC sterile 1∶1 segregating line (AMS) were used. Characteristics of all of the materials are shown in Table 3.

**Table 3:** Materials of *B. napus* and *B. rapa*.

Name	Source	Species	Whorl 2	Whorl 3
WTc	Wild type of *B. rapa*	*B. rapa* subsp. *chinensis* (AA)	Petal	Stamen
HGMSb	Fertile plants in HGMS		Petal	Stamen
HGMSa	SC sterile plants in HGMS		Sepal	Carpel
HGMSIIa	SC sterile plants in HGMSII		Sepaloid	Carpelloid
HGMSIIb	Fertile plants in HGMSII		Petal	Stamen
HGMSb2	Another maintainer of HGMSa		Sepal	Stamen
WTn	Wild type of *B. napus*	*B. napus* (AACC)	Petal	Stamen
Apt	Apetalous mutant of *B. napus*		Apetalous	Stamen
AMSb	Fertile plants in AMS		Apetalous	Stamen
AMSa	SC sterile plants in AMS		Apetalous	Carpel

Note: Petal and stamen phenotypes were identified according to their morphological characteristics and appearance as observed by scanning electron microscope.

### Scanning electron microscopy (SEM)

Petals were picked from the flower buds, fixed with 3% glutaraldehyde at 4°C overnight, washed with 0.025 M PBS (phosphate buffered saline, pH 7.0) four times and incubated for 4 h in 1% osmic acid. Samples were washed again in 0.05 M PBS, dehydrated gradually in an ethanol series, dried in liquid carbon dioxide, sputter-coated with gold palladium, and observed with a JEOL JSM-6360LV scanning electron microscope.

### Genetic analysis

After crossing the four selfing lines of *B. rapa* (Aj, Lx, ez1 and ey5) with HGMSa as well as the three selfing lines of *B. napus* (S3B, S4B and K407) with AMSa, the segregation of fertile versus SC sterile plants in each population of BC_1_ and F_2_ was examined.

### RNA and DNA extraction and gene isolation

For each 1∶1 segregating line, fertile plants and SC sterile plants were identified and separated into two parts at the early stage of floral bud development, and for each of them, total RNA was extracted from its small younger floral buds using the RNAiso Reagent (TaKaRa). First-strand cDNA was synthesized by Prime Script RT-PCR kit (TaKaRa). At the same time, the genomic DNA of these materials was extracted.

### Expression and isolation of *AP3* genes

The *AP3* gene-specific primers AP3-F (ATGGCGAGAGGGAAGATCCA) and AP3-R (TTATTCAAGAAGGTGGAAGGTAATGAT) were designed according to the sequences of *AP3* genes (DQ372719, AY623003) from *B. napus* and *B. rapa*. The expression analysis of *AP3* genes was conducted using reverse transcriptase polymerase chain reaction (RT-PCR). The amounts of templates were carefully adjusted to match that of the control gene, the cytosolic *18S* rRNA gene. Thirty-cycle RT-PCR was performed using AP3-F and AP3-R primers at the 61°C annealing temperature. PCR products were finally fractionated in 2.5% agarose for 4.5 h and digitally photographed. At the same time, the amplified *AP3* gene fragments (about 675–800 bp) were excised and purified using an AxyPrep™ DNA Gel Extraction Kit (Axygen), cloned into the pGEM-T Easy Vector (Promega), and sequenced by Beijing Sunbiotech Co. Ltd. Similarly, full-length DNA sequences of *AP3* genes were isolated.

To classify the two kinds of *AP3* genes we had cloned, the special primers 24-F (AGAACCAGACCAACCGACAA), 24-R (CGTAAGCACGTGATCCTTCG) and R670 (AAAGTGTTTTCCATTTCTTCCTCAA) were designed. With the 24-F and 24-R primers, one kind of *AP3* gene that contains the specific 24-bp sequence could be easily isolated.

### Intercross analysis

To intercross AMS with HGMS and HGMSII, the four combinations AMSa×HGMSb, HGMSa×AMSb, AMSa×HGMSIIb and HGMSIIa×AMSb were generated artificially and sown in the field. Fertile plants and SC sterile plants in each combination were identified at the early stage of floral bud development, and their small younger buds were used for RNA extraction. During flowering, fertile and SC sterile plants were investigated.

## Supporting Information

Figure S1 **Nucleotide alignment of** ***BraA.AP3.a*** **and** ***BraA.AP3.b*** **of** ***B.rapa*** **.**
(DOC)

Figure S2 **Amino acid alignment of** ***BraA.AP3.a*** **and** ***BraA.AP3.b*** **of** ***B.rapa*** **.**
(DOC)

Figure S3 **Partial** ***AP3*** **genes alignment between wild type and SC mutant HGMS of** ***B.rapa*** **.**
(DOC)

Figure S4 **Nucleotide alignment of** ***B.AP3.b*** **among** ***B.rapa*** **and** ***B.oleracea*** **and** ***B.napus*** **.**
(DOC)

Figure S5 **Amino acid alignment of** ***B.AP3.a*** **and** ***B.AP3.b*** **among** ***B.rapa*** **and** ***B.oleracea*** **and** ***B.napus*** **.**
(DOC)

Figure S6 **Nucleotide alignment of** ***B.AP3.a*** **among** ***B.rapa*** **and** ***B.oleracea*** **and** ***B.napus*** **.**
(DOC)

Figure S7 **Flowers and** ***AP3*** **genes distribution of F_1_ hybrids between AMS and HGMSII.** (A) Flowers and petals of F_1_ hybrids between AMS and HGMSII. Fertile plant and SC male sterile plant in HGMSIIa×AMSb were named HAIIb and HAIIa, in AMSa×HGMSIIb were named AHIIb and AHIIa; (B) DNA amplification from HAIIb, HAIIa, AHIIb and AHIIa using 24-F and 24-R primers. Fertile plants AHIIb contained a *B.AP3.a* gene from male parent HGMSIIb, and their petals and stamens developed normally; (C) DNA amplification from HAb and HAa using AP3-F and AP3-R primers. Fertile plants HAIIb had a *BnaA.AP3.b* gene from AMSb, their stamens developed normally while petals showed sepaloid identity.
(DOC)

Table S1 **Segregation of SC sterile lines HGMS and AMS by inter-sibling and self-crossings.**
(DOC)
