# Supplementary material for: Functional Analysis of the Two Brassica AP3 Genes Involved in Apetalous and Stamen Carpelloid Phenotypes
Source: PLoS One. 2011 Jun 30;6(6):e20930. doi: 10.1371/journal.pone.0020930 (PMC3128040; doi:10.1371/journal.pone.0020930)
Supplement: Figure S1 — Nucleotide alignment of BraA.AP3.a and BraA.AP3.b of B.rapa . (DOC) [file pone.0020930.s001.doc]

Bra007067 (1) ATGGCGAGAGGGAAGATCCAGATCAAGAGGATAGAGAACCAGACCAACCGACAAGTAACGTATTCCAAGA

*BraA.AP3.a* (1) ATGGCGAGAGGGAAGATCCAGATCAAGAGGATAGAGAACCAGACCAACCGACAAGTAACATATTCCAAGA

Bra014822 (1) ATGGCGAGAGGGAAGATCCAGATCAAGAGGATAGAGAACCAGACAAACCGACAAGTGACGTATTCCAAGA

*BraA.AP3.b* (1) ATGGCGAGAGGGAAGATCCAGATCAAGAGGATAGAGAACCAGACAAACCGACAAGTGACGTATTCCAAGA

Bra007067 (71) GAAGAAATGGTCTGTTCAAGAAAGCTCACGAGCTTACGGTTTTGTGTGATGCTAGGGTTTCGATTATCAT

*BraA.AP3.a* (71) GAAGAAATGGTTTGTTCAAGAAAGCTCACGAGCTTACGGTTTTGTGTGATGCTAGGGTTTCGATTATCAT

Bra014822 (71) GAAGAAATGGTTTGTTCAAGAAAGCTCACGAGCTCACGGTTTTGTGTGACGCTAGGGTTTCGATTATCAT

*BraA.AP3.b* (71) GAAGAAATGGTTTGTTCAAGAAAGCTCACGAGCTCACGGTTTTGTGTGACGCTAGGGTTTCGATTATCAT

Bra007067 (141) GTTCTCTAGCTCTAACAAGCTTCATGAGTTCATTAGCCCTAACACCACAACAAAGGAGATCATAGATCTG

*BraA.AP3.a* (141) GTTCTCTAGCTCTAACAAGCTTCATGAGTTCATTAGCCCTAACACCACAACAAAGGAGATCATAGATCTG

Bra014822 (141) GTTCTCTAGTTCCAACAAGCTTCATGAGTTTATCAGCCCTAACACCACAACGAAGGAGATCATAGATCTG

*BraA.AP3.b* (141) GTTCTCTAGTTCCAACAAGCTTCATGAGTTTATCAGCCCTAACACCACAACGAAGGAGATCATAGATCTG

Bra007067 (211) TACCAAACCGTTTCTGATGTTGATGTTTGGAGCGCACACTATGAGAGAATGCAAGAAACCAAGAGGAAGC

*BraA.AP3.a* (211) TACCAAACCGTTTCCGATGTTGATGTTTGGAGCGCACACTATGAGAGAATGCAAGAAACCAAGAGGAAGC

Bra014822 (211) TACCAAACAGTTTCTGATGTTGATGTTTGGAGTGCTCACTATGAGAGAATGCAAGAAACCAAGAGGAAAT

*BraA.AP3.b* (211) TACCAAACAGTTTCTGATGTTGATGTTTGGAGTGCTCACTATGAGAGAATGCAAGAAACCAAGAGGAAAT

Bra007067 (281) TGTTGGAGACAAATAGAAAGCTTCGGACTCAGATTAAGCAGAGGCTAGGTGAGTGTTTGGACGAACTTGA

*BraA.AP3.a* (281) TGTTGGAGACAAATAGAAAGCTCCGGACTCAGATTAAGCAGAGGCTAGGTGAGTGTTTGGACGAACTTGA

Bra014822 (281) TATTGGAGACAAATAGAAATCTTCGGACTCAGATTAAACAGAGGCTAGGTGAGTGTTTAGACGAGCTTGA

*BraA.AP3.b* (281) TATTGGAGACAAATAGAAATCTTCGGACTCAGATTAAACAGAGGCTAGGTGAGTGTTTAGACGAGCTTGA

Bra007067 (351) TATTCAGGAGCTGCGTAGTCTTGAGGAAGAAATGGAAAACACTTTCAAACTCGTGCGTGAGCGCAAGTTT

*BraA.AP3.a* (351) TATTCAGGAGCTTCGTAGTCTTGAGGAAGAAATGGAAAACACTTTCAAACTCGTTCGTGAGCGCAAGTTT

Bra014822 (351) TATTCAGGAGCTGCGTAGTCTTGAGGAAGAAATGGAAAACACTTTCAAACTCGTTCGCGAGCGCAAGTTT

*BraA.AP3.b* (351) TATTCAGGAGCTGCGTAGTCTTGAGGAAGAAATGGAAAACACTTTCAAACTCGTTCGCGAGCGCAAGTTT

Bra007067 (421) AAATCCCTTGGAAATCAGATCGAAACCACCAAGAAAAAGAACAAAAGTCAACAAGACATACAAAAGAATC

*BraA.AP3.a* (421) AAATCCCTTGGGAATCAGATCGAAACCACCAAGAAAAAGAACAAAAGTCAACAAGACATACAAAAGAATC

Bra014822 (421) AAATCACTTGGGAACCAAATCGAGACCACCAAGAAAAAGAACAAGAGTCAACAAGACATACAAAAGAATC

*BraA.AP3.b* (421) AAATCACTTGGGAACCAAATCGAGACCACCAAGAAAAAGAACAAGAGTCAACAAGACATACAAAAGAATC

Bra007067 (491) TCATACATGAGCTGGAGCTAAGGGCAGAAGATCCTCACTATGGCCTAGTAGACAATGGAGGCGACTACGA

*BraA.AP3.a* (491) TCATACATGAGCTGGAGCTAAGAGCAGAAGATCCTCACTATGGCCTAGTAGACAATGGAGGCGACTACGA

Bra014822 (491) TCATACATGAGCTGGAACTAAGAGCAGAAGATCCTCATTATGGACTAGTAGAAAATGGAGGAGACTACGA

*BraA.AP3.b* 491) TCATACATGAGCTGGAACTAAGAGCAGAAGATCCTCATTATGGACTAGTAGACAATGGAGGAGACTACGA

Bra007067 (561) TTCGGTTCTTGGATATCAAATCGAAGGATCACGTGCTTACGCTCTTCGTTACCATCAGAACCATCATCAC

*BraA.AP3.a* (561) TTCGGTTCTTGGATATCAAATCGAAGGATCACGTGCTTACGCTCTTCGTTACCATCAGAACCATCATCAC

Bra014822 (561) TTCAGTTCTTGGATATCAA------------------------CTTCGCTTCCATCAGAACCATCACCAC

*BraA.AP3.b* (561) TTCAGTTCTTGGATATCAA------------------------CTTCGCTTCCATCAGAACCATCACCAC

Bra007067 (631) CATTACCCCAACCATGCCCTTCATGCACCATCTGCCTCTGACATCATTACCTTCCACCTTCTCGAATGA

*BraA.AP3.a* (631) CATTACCCCAACCATACCCTTCATGCACCATCTGCCTCTGACATCATTACCTTCCACCTTCTTGAATAA

Bra014822 (607) CATTACCCCAACCATGCCCTTCATGCAGCATCTGCCTCTGATATCATTACCTTCCACCTTCTTGAATAA

*BraA.AP3.b* (607) CATTACCCCAACCATGCCCTTCATGCAGCATCTGCCTCTGATATCATTACCTTCCACCTTCTTGAATAA

**Figure S1. Nucleotide alignment of *BraA.AP3.a* and *BraA.AP3.b* of *B.rapa*.**

Note：Bra007067 and Bra014822 are two *AP3* genes from the *B.rapa* genome database (http://brassicadb.org.brad/).
