# Supplementary material for: Functional Analysis of the Two Brassica AP3 Genes Involved in Apetalous and Stamen Carpelloid Phenotypes
Source: PLoS One. 2011 Jun 30;6(6):e20930. doi: 10.1371/journal.pone.0020930 (PMC3128040; doi:10.1371/journal.pone.0020930)
Supplement: Figure S2 — Amino acid alignment of BraA.AP3.a and BraA.AP3.b of B.rapa . (DOC) [file pone.0020930.s002.doc]

*AP3-Arab* (1) MARGKIQIKRIENQTNRQVTYSKRRNGLFKKAHELTVLCDARVSIIMFSSSNKLHEYISPNTTTKEIVDL

Bra007067 (1) MARGKIQIKRIENQTNRQVTYSKRRNGLFKKAHELTVLCDARVSIIMFSSSNKLHEFISPNTTTKEIIDL

*BraA.AP3.a* (1) MARGKIQIKRIENQTNRQVTYSKRRNGLFKKAHELTVLCDARVSIIMFSSSNKLHEFISPNTTTKEIIDL

Bra014822 (1) MARGKIQIKRIENQTNRQVTYSKRRNGLFKKAHELTVLCDARVSIIMFSSSNKLHEFISPNTTTKEIIDL

*BraA.AP3.b* (1) MARGKIQIKRIENQTNRQVTYSKRRNGLFKKAHELTVLCDARVSIIMFSSSNKLHEFISPNTTTKEIIDL

*AP3-Arab* (71) YQTISDVDVWATQYERMQETKRKLLETNRNLRTQIKQRLGECLDELDIQELRRLEDEMENTFKLVRERKF

Bra007067 (71) YQTVSDVDVWSAHYERMQETKRKLLETNRKLRTQIKQRLGECLDELDIQELRSLEEEMENTFKLVRERKF

*BraA.AP3.a* (71) YQTVSDVDVWSAHYERMQETKRKLLETNRKLRTQIKQRLGECLDELDIQELRSLEEEMENTFKLVRERKF

Bra014822 (71) YQTVSDVDVWSAHYERMQETKRKLLETNRNLRTQIKQRLGECLDELDIQELRSLEEEMENTFKLVRERKF

*BraA.AP3.b* (71) YQTVSDVDVWSAHYERMQETKRKLLETNRNLRTQIKQRLGECLDELDIQELRSLEEEMENTFKLVRERKF

*AP3-Arab* (141) KSLGNQIETTKKKNKSQQDIQKNLIHELELRAEDPHYGLVDNGGDYDSVLGYQIEGSRAYALRFHQNHHH

Bra007067 (141) KSLGNQIETTKKKNKSQQDIQKNLIHELELRAEDPHYGLVDNGGDYDSVLGYQIEGSRAYALRYHQNHHH

*BraA.AP3.a* (141) KSLGNQIETTKKKNKSQQDIQKNLIHELELRAEDPHYGLVDNGGDYDSVLGYQIEGSRAYALRYHQNHHH

Bra014822 (141) KSLGNQIETTKKKNKSQQDIQKNLIHELELRAEDPHYGLVENGGDYDSVLGYQ--------LRFHQNHHH

*BraA.AP3.b* (141) KSLGNQIETTKKKNKSQQDIQKNLIHELELRAEDPHYGLVDNGGDYDSVLGYQ--------LRFHQNHHH

*AP3-Arab* (211) YYPNHGLHAPSASDIITFHLLE

Bra007067 (211) HYPNHALHAPSASDIITFHLLE

*BraA.AP3.a* (211) HYPNHALHAPSASDIITFHLLE

Bra014822 (203) HYPNHALHAASASDIITFHLLE

*BraA.AP3.b* (203) HYPNHALHAASASDIITFHLLE

**Figure S2. Amino acid alignment of *BraA.AP3.a* and *BraA.AP3.b* of *B.rapa*.**

Note：*AP3-Arab*: *AP3* gene of *Arabidopsis*. Bra007067 and Bra014822 are two *AP3* genes from the *B.rapa* genome database (http://brassicadb.org.brad/).
