# Supplementary material for: Functional Analysis of the Two Brassica AP3 Genes Involved in Apetalous and Stamen Carpelloid Phenotypes
Source: PLoS One. 2011 Jun 30;6(6):e20930. doi: 10.1371/journal.pone.0020930 (PMC3128040; doi:10.1371/journal.pone.0020930)
Supplement: Figure S3 — Partial AP3 genes alignment between wild type and SC mutant HGMS of B.rapa . (DOC) [file pone.0020930.s003.doc]

**AP3-F primer** **24-F primer**

ATGGCGAGAGGGAAGATCCA AGAACCAGACCAACCGACAA

*BraA.AP3.a* DNA (1) ATGGCGAGAGGGAAGATCCAGATCAAGAGGATAGAGAACCAGACCAACCGACAAGTAACGTATTCCAAGA

*BraA.AP3.a-16* DNA (1) ATGGCGAGAGGGAAGATCCAGATCAAGAGGATAGA----------------CAAGTAACGTATTCCAAGA

*BraA.AP3.a* cDNA (1) ATGGCGAGAGGGAAGATCCAGATCAAGAGGATAGAGAACCAGACCAACCGACAAGTAACGTATTCCAAGA

*BraA.AP3.b* DNA (1) ATGGCGAGAGGGAAGATCCAGATCAAGAGGATAGAGAACCAGACCAACCGACAAGTGACGTATTCCAAGA

*BraA.AP3.b+51* DNA (1) ATGGCGAGAGGGAAGATCCAGATCAAGAGGATAGAGAACCAGACCAACCGACAAGTGACGTATTCCAAGA

*BraA.AP3.b* cDNA (1) ATGGCGAGAGGGAAGATCCAGATCAAGAGGATAGAGAACCAGACCAACCGACAAGTGACGTATTCCAAGA

*BraA.AP3.a* DNA (71) GAAGAAATGGTCTGTTCAAGAAAGCTCACGAGCTTACGGTTTTGTGTGATGCTAGGGTTTCGATTATCAT

*BraA.AP3.a-16* DNA (55) GAAGAAATGGTCTGTTCAAGAAAGCTCACGAGCTTACGGTTTTGTGTGATGCTAGGGTTTCGATTATCAT

*BraA.AP3.a* cDNA (71) GAAGAAATGGTCTGTTCAAGAAAGCTCACGAGCTTACGGTTTTGTGTGATGCTAGGGTTTCGATTATCAT

*BraA.AP3.b* DNA (71) GAAGAAATGGTTTGTTCAAGAAAGCTCACGAGCTCACGGTTTTGTGTGACGCTAGGGTTTCGATTATCAT

*BraA.AP3.b+51* DNA (71) GAAGAAATGGTTTGTTCAAGAAAGCTCACGAGCTCACGGTTTTGTGTGACGCTAGGGTTTCGATTATCAT

*BraA.AP3.b* cDNA (71) GAAGAAATGGTTTGTTCAAGAAAGCTCACGAGCTCACGGTTTTGTGTGACGCTAGGGTTTCGATTATCAT

*BraA.AP3.a* DNA (141) GTTCTCTAGCTCTAACAAGCTTCATGAGTTCATTAGCCCTAACACCACGTACATAATCTCTCTAAAACTA

*BraA.AP3.a-16* DNA (125) GTTCTCTAGCTCTAACAAGCTTCATGAGTTCATTAGCCCTAACACCACGTACATAATCTCTCTAAAACTA

*BraA.AP3.a* cDNA (141) GTTCTCTAGCTCTAACAAGCTTCATGAGTTCATTAGCCCTAACACCAC----------------------

*BraA.AP3.b* DNA (141) GTTCTCTAGTTCCAACAAGCTTCATGAGTTTATCAGCCCTAACACCACGTAGAGAATATCTATCTTAAAC

*BraA.AP3.b+51* DNA (141) GTTCTCTAGTTCCAACAAGCTTCATGAGTTTATCAGCCCTAACACCACGTAGAGAATATCTATCTTAAAC

*BraA.AP3.b* cDNA (141) GTTCTCTAGTTCCAACAAGCTTCATGAGTTTATCAGCCCTAACACCAC----------------------

*BraA.AP3.a* DNA (211) AATTTTAAATGCTTTA------------------------------------------------------

*BraA.AP3.a-16* DNA (195) AATTTTAAATGCTTTA------------------------------------------------------

*BraA.AP3.a* cDNA (189) ----------------------------------------------------------------------

*BraA.AP3.b* DNA (211) ACCACTCTTATATATA---------------------------------------------------TTT

*BraA.AP3.b+51* DNA (211) ACCACTCTTATATATATATATATATATATATATATATATATATATATATATATATATAAATATTTCTTTT

*BraA.AP3.b* cDNA (189) ----------------------------------------------------------------------

*BraA.AP3.a* DNA (227) ----CTTTTCTTCTAGTTAATGACTTTTTGTGTTTTGTTTTGTTGGTTATAGAACAAAGGAGATCATAGA

*BraA.AP3.a-16* DNA (211) ----CTTTTCTTCTAGTTAATGACTTTTTGTGTTTTGTTTTGTTGGTTATAGAACAAAGGAGATCATAGA

*BraA.AP3.a* cDNA (189) ----------------------------------------------------AACAAAGGAGATCATAGA

*BraA.AP3.b* DNA (230) CTTTTCTTATAGTTAACTACTTTTTAATTTGTGTGGTCTTGTTGGTTTATAGAACGAAGGAGATCATAGA

*BraA.AP3.b+51* DNA (281) CTTTTCTTATAGTTAACTACTTTTTAATTTGTGTGGTCTTGTTGGTTTATAGAACGAAGGAGATCATAGA

*BraA.AP3.b* cDNA (189) ----------------------------------------------------AACGAAGGAGATCATAGA

*BraA.AP3.a* DNA (293) TCTGTACCAAACCGTTTCTGATGTTGATGTTTGGAGCGCACACTATGAGGTT---TTCTTCTTCTTAGAT

*BraA.AP3.a-16* DNA (277) TCTGTACCAAACCGTTTCTGATGTTGATGTTTGGAGCGCACACTATGAGGTT---TTCTTCTTCTTAGAT

*BraA.AP3.a* cDNA (207) TCTGTACCAAACCGTTTCTGATGTTGATGTTTGGAGCGCACACTATGAG---------------------

*BraA.AP3.b* DNA (300) TCTGTACCAAACAGTTTCTGATGTTGATGTTTGGAGTGCTCACTATGAGGTTCTTTTCTTCTTCTTAGAT

*BraA.AP3.b+51* DNA (351) TCTGTACCAAACAGTTTCTGATGTTGATGTTTGGAGTGCTCACTATGAGGTTCTTTTCTTCTTCTTAGAT

*BraA.AP3.b* cDNA (207) TCTGTACCAAACAGTTTCTGATGTTGATGTTTGGAGTGCTCACTATGAG---------------------

*BraA.AP3.a* DNA (360) CTTGATTCTTGTTCTCGACATGTGTGTTTTTTTTGGTTTGGTGAATTTTGGTTATTCGTTTTTGTAGAGA

*BraA.AP3.a-16* DNA (344) CTTGATTCTTGTTCTCGACATGTGTGTTTTTTTTGGTTTGGTGAATTTTGGTTATTCGTTTTTGTAGAGA

*BraA.AP3.a* cDNA (256) -------------------------------------------------------------------AGA

*BraA.AP3.b* DNA (370) CCTCTTATTCTTGTTCTTGATATGTGTTTCTTGATGGTTTGGTGAAATTGGTGAGTCTTTGTTGTAGAGA

*BraA.AP3.b+51* DNA (421) CCTCTTATTCTTGTTCTTGATATGTGTTTCTTGATGGTTTGGTGAAATTGGTGAGTCTTTGTTGTAGAGA

*BraA.AP3.b* cDNA (256) -------------------------------------------------------------------AGA

*BraA.AP3.a* DNA (430) ATGCAAGAAACCAAGAGGAAGCTGTTGGAGACAAATAGAAAGCTTCGGACTCAGATTAAGTATGTGTTTC

*BraA.AP3.a-16* DNA (414) ATGCAAGAAACCAAGAGGAAGCTGTTGGAGACAAATAGAAAGCTTCGGACTCAGATTAAGTATGTGTTTC

*BraA.AP3.a* cDNA (259) ATGCAAGAAACCAAGAGGAAGCTGTTGGAGACAAATAGAAAGCTTCGGACTCAGATTAA-----------

*BraA.AP3.b* DNA (440) ATGCAAGAAACCAAGAGGAAATTATTGGAGACAAATAGAAATCTTCGGACTCAGATTAAGTATTTGTTTC

*BraA.AP3.b+51* DNA (491) ATGCAAGAAACCAAGAGGAAATTATTGGAGACAAATAGAAATCTTCGGACTCAGATTAAGTATTTGTTTC

*BraA.AP3.b* cDNA (259) ATGCAAGAAACCAAGAGGAAATTATTGGAGACAAATAGAAATCTTCGGACTCAGATTAA-----------

*BraA.AP3.a* DNA (500) TTTCTCT-----------TCTCTCATCATCTCTCTCTAAACTTAAAAATCAAATTATAAAATTATAAACT

*BraA.AP3.a-16* DNA (484) TTTCTCT-----------TCTCTCATCATCTCTCTCTAAACTTAAAAATCAAATTATAAAATTATAAACT

*BraA.AP3.a* cDNA (318) ----------------------------------------------------------------------

*BraA.AP3.b* DNA (510) CTGTTCTCTTCTCCCATATCTCTCTCTTTAAACTTAAAAAATTACGAAATTACGAAATACTAACAAACTT

*BraA.AP3.b+51* DNA (561) CTGTTCTCTTCTCCCATATCTCTCTCTTTAAACTTAAAAAATTACGAAATTACGAAATACTAACAAACTT

*BraA.AP3.b* cDNA (318) ----------------------------------------------------------------------

*BraA.AP3.a* DNA (559) TTTGTTATCAATTGTAGGCAGAGGCTAGGTGAGTGTTTGGACGAACTTGATATTCAGGAGCTGCGTAGTC

*BraA.AP3.a-16* DNA (543) TTTGTTATCAATTGTAGGCAGAGGCTAGGTGAGTGTTTGGACGAACTTGATATTCAGGAGCTGCGTAGTC

*BraA.AP3.a* cDNA (318) -----------------GCAGAGGCTAGGTGAGTGTTTGGACGAACTTGATATTCAGGAGCTGCGTAGTC

*BraA.AP3.b* DNA (580) TGGTTTATTAATTGTAGACAGAGGCTAGGTGAGTGTTTAGACGAGCTTGATATTCAGGAGCTGCGTAGTC

*BraA.AP3.b+51* DNA (631) TGGTTTATTAATTGTAGACAGAGGCTAGGTGAGTGTTTAGACGAGCTTGATATTCAGGAGCTGCGTAGTC

*BraA.AP3.b* cDNA (318) -----------------ACAGAGGCTAGGTGAGTGTTTAGACGAGCTTGATATTCAGGAGCTGCGTAGTC

*BraA.AP3.a* DNA (629) TTGAGGAAGAAATGGAAAACACTTT

*BraA.AP3.a-16* DNA (613) TTGAGGAAGAAATGGAAAACACTTT

*BraA.AP3.a* cDNA (371) TTGAGGAAGAAATGGAAAACACTTT

*BraA.AP3.b* DNA (650) TTGAGGAAGAAATGGAAAACACTTT

*BraA.AP3.b+51* DNA (701) TTGAGGAAGAAATGGAAAACACTTT

*BraA.AP3.b* cDNA (371) TTGAGGAAGAAATGGAAAACACTTT

TTGAGGAAGAAATGGAAAACACTTT

**R670 primer**

**Figure S3. Partial *AP3* genes alignment between wild type and SC mutant HGMS of *B.rapa.***

Note: *BraA.AP3.a* and *BraA.AP3.b* were the two *AP3* genes of wild type *B. rapa*. *BraA.AP3.a-16* and *BraA.AP3.b+51* were the two *AP3* genes of SC mutant HGMS, and their mutational sites were showed by arrows.
