# Supplementary material for: Functional Analysis of the Two Brassica AP3 Genes Involved in Apetalous and Stamen Carpelloid Phenotypes
Source: PLoS One. 2011 Jun 30;6(6):e20930. doi: 10.1371/journal.pone.0020930 (PMC3128040; doi:10.1371/journal.pone.0020930)
Supplement: Figure S4 — Nucleotide alignment of B.AP3.b among B.rapa and B.oleracea and B.napus . (DOC) [file pone.0020930.s004.doc]

*BraA.AP3.b* (1) ATGGCGAGAGGGAAGATCCAGATCAAGAGGATAGAGAACCAGACAAACCGACAAGTGACGTATTCCAAGAGAAGAAATGGTTTGTTCAAG

*BnaA.AP3.b*  (1) ............................................A....................C.............G..........

*BnaC.AP3.b* (1) ............................................C....................A.............G..........

*BnaC.AP3.b-Mu*  (1) ............................................C....................A.............T..........

*BolC.AP3.b* (1) ............................................C....................A.............G..........

*BraA.AP3.b* (91) AAAGCTCACGAGCTCACGGTTTTGTGTGACGCTAGGGTTTCGATTATCATGTTCTCTAGTTCCAACAAGCTTCATGAGTTTATCAGCCCT

*BnaA.AP3.b* (91) ....................T......................................T..............................

*BnaC.AP3.b* (91) ....................C......................................C..............................

*BnaC.AP3.b-Mu*  (91) ....................C......................................C..............................

*BolC.AP3.b* (91) ....................C......................................C..............................

*BraA.AP3.b* (181) AACACCACAACGAAGGAGATCATAGATCTGTACCAAACAGTTTCTGATGTTGATGTTTGGAGTGCTCACTATGAGAGAATGCAAGAAACC

*BnaA.AP3.b*  (181) .....................A........................................T...........................

*BnaC.AP3.b*  (181) .....................T........................................C...........................

*BnaC.AP3.b-Mu*(181) .....................T........................................C...........................

*BolC.AP3.b* (181) .....................T........................................C...........................

*BraA.AP3.b* (271) AAGAGGAAATTATTGGAGACAAATAGAAATCTTCGGACTCAGATTAAACAGAGGCTAGGTGAGTGTTTAGACGAGCTTGATATTCAGGAG

*BnaA.AP3.b* (271) .........T.AT..............................................T........A......C..............

*BnaC.AP3.b* (271) .........C.GC..............................................C........G......T..............

*BnaC.AP3.b-Mu*(271) .........C.GC..............................................C........G......T..............

*BolC.AP3.b*  (271) .........C.GC..............................................C........G......T..............

*BraA.AP3.b* (361) CTGCGTAGTCTTGAGGAAGAAATGGAAAACACTTTCAAACTCGTTCGCGAGCGCAAGTTTAAATCACTTGGGAACCAAATCGAGACCACC

*BnaA.AP3.b*  (361) ....G............................................................A........C...............

*BnaC.AP3.b*  (361) ....G............................................................G........T...............

*BnaC.AP3.b-Mu* (361) ....G............................................................G........T...............

*BolC.AP3.b* (361) ....T............................................................G........T...............

*BraA.AP3.b* (451) AAGAAAAAGAACAAGAGTCAACAAGACATACAAAAGAATCTCATACATGAGCTGGAACTAAGAGCAGAAGATCCTCATTATGGACTAGTA

*BnaA.AP3.b* (451) .................T..A.....................................................................

*BnaC.AP3.b*  (451) .................C..G.....................................................................

*BnaC.AP3.b-Mu*  ­(451) .................C..G.....................................................................

*BolC.AP3.b* (451) .................C..G.....................................................................

*BraA.AP3.b* (541) GACAATGGAGGAGACTACGATTCAGTTCTTGGATATCAACTTCGCTTCCATCAGAACCATCACCACCATTACCCCAACCATGCCCTTCAT

*BnaA.AP3.b* (541) .......................A....................C..........................C..................

*BnaC.AP3.b* (541) .......................C....................T..........................T..................

*BnaC.AP3.b-Mu* (541) .......................C....................T..........................T..................

*BolC.AP3.b*  (541) .......................C....................T..........................T..................

*BraA.AP3.b*  (631) GCAGCATCTGCCTCTGATATCATTACCTTCCACCTTCTTGAATAA

*BnaA.AP3.b* (631) .C...............T....................T......

*BnaC.AP3.b* (631) .A...............C....................T......

*BnaC.AP3.b-Mu* (631) .A...............C....................T......

*BolC.AP3.b* (631) .A...............C....................C......

**Figure S4. Nucleotide alignment of *B.AP3.b* among *B.rapa* and *B.oleracea* and *B.napus*.**

Note：*BraA.AP3.b*: *B.rapa*; *BolC.AP3.b*: *B.oleracea*; *BnaA.AP3.b* and *BnaC.AP3.b*: *B. napus*; *BnaC.AP3.b-Mu*: *BnaC.AP3.b* with a single nucleotide mutation (shown by an arrow).
