# Supplementary material for: Functional Analysis of the Two Brassica AP3 Genes Involved in Apetalous and Stamen Carpelloid Phenotypes
Source: PLoS One. 2011 Jun 30;6(6):e20930. doi: 10.1371/journal.pone.0020930 (PMC3128040; doi:10.1371/journal.pone.0020930)
Supplement: Figure S5 — Amino acid alignment of B.AP3.a and B.AP3.b among B.rapa and B.oleracea and B.napus . (DOC) [file pone.0020930.s005.doc]

*BraA.AP3.a* (1) MARGKIQIKRIENQTNRQVTYSKRRNGLFKKAHELTVLCDARVSIIMFSSSNKLHEFISPNTTTKEIIDLYQTVSDVDVW

*BnaA.AP3.a* (1) ..........................G........................................I............

*BnaC.AP3.a*  (1) ..........................G........................................I............

D5 (1) ..........................G........................................I............

*BolC.AP3.a* (1) ..........................G........................................I............

*BraA.AP3.b* (1) ..........................G........................................I............

*BnaA.AP3.b* (1) ..........................G........................................I............

*BnaC.AP3.b* (1) ..........................G........................................L............

*BnaC.AP3.b_-Mn_*  (1) ..........................V........................................L............

*BolC.AP3.b*  (1) ..........................G........................................L............

*BraA.AP3.a* (81) SAHYERMQETKRKLLETNRKLRTQIKQRLGECLDELDIQELRSLEEEMENTFKLVRERKFKSLGNQIETTKKKNKSQQDI

*BnaA.AP3.a* (81) ...................................L.....R......................................

*BnaC.AP3.a*  (81) ...................................L.....R......................................

D5 (81) ...................................L.....R......................................

*BolC.AP3.a* (81) ...................................L.....R......................................

*BraA.AP3.b* (81) ...................................L.....R......................................

*BnaA.AP3.b* (81) ...................................L.....R......................................

*BnaC.AP3.b* (81) ...................................F.....R......................................

*BnaC.AP3.b_-Mn_*  (81) ...................................F.....R......................................

*BolC.AP3.b* (81) ...................................F.....L......................................

*BraA.AP3.a*  (161) QKNLIHELELRAEDPHYGLVDNGGDYDSVLGYQ**IEGSRAYA**LRYHQNHHHHYPNHALHAPSASDIITFHLLE

*BnaA.AP3.a* (161) .................................**IEGSRAYA**......H.......T..A.............

*BnaC.AP3.a* (161) .................................**IEGSRAYA**......Q.......A..A.............

D5 (161) .................................**IEGSRAYA**......Q.......A..A.............

*BolC.AP3.a* (161) .................................**IEGSRAYA**......H.......A..A.............

*BraA.AP3.b* (161) .................................**--------**......H.......A..A.............

*BnaA.AP3.b* (161) .................................**--------**......H.......A..A.............

*BnaC.AP3.b* (161) .................................**--------**......H.......A..E.............

*BnaC.AP3.b_-Mn_* (161) .................................**--------**......H.......A..E.............

*BolC.AP3.b* (161) .................................**--------**......H.......A..E.............

**Figure S5. Amino acid alignment of *B.AP3.a* and *B.AP3.b* among *B.rapa* and *B.oleracea* and *B.napus*.**

Note: *BraA.AP3.a* and *BraA.AP3.b*: *B.rapa*; *BolC.AP3.a* and *BolC.AP3.b*: *B.oleracea*; *BnaA.AP3.a* and *BnaA.AP3.b* and *BnaC.AP3.a* and *BnaC.AP3.b*: *B. napus*; D5: *BnaC.AP3.a* of Apt, AMSb and AMSa with an 86-bp foreign insertion (the foreign sequence be deleted); *BnaC.AP3.b_-Mu_*: *BnoAP3_-24_* of Apt, AMSb and AMSa with a single amino acid mutation (shown by an arrow).
