# Supplementary material for: Functional Analysis of the Two Brassica AP3 Genes Involved in Apetalous and Stamen Carpelloid Phenotypes
Source: PLoS One. 2011 Jun 30;6(6):e20930. doi: 10.1371/journal.pone.0020930 (PMC3128040; doi:10.1371/journal.pone.0020930)
Supplement: Figure S6 — Nucleotide alignment of B.AP3.a among B.rapa and B.oleracea and B.napus . (DOC) [file pone.0020930.s006.doc]

*BraA.AP3.a*  (1) ATGGCGAGAGGGAAGATCCAGATCAAGAGGATAGAGAACCAGACCAACCGACAAGTAACGTATTCCAAGAGAAGAAATGGTCTGTTCAAG

*BnaA.AP3.a* (1) ........................................................A..A.....C...............T........

*BnaC.AP3.a* (1) ........................................................G..G.....T...............T........

D5 (1) ........................................................G..G.....T...............T........

*BolC.AP3.a* (1) ........................................................G..G.....T...............T........

*BraA.AP3.a* (91) AAAGCTCACGAGCTTACGGTTTTGTGTGATGCTAGGGTTTCGATTATCATGTTCTCTAGCTCTAACAAGCTTCATGAGTTCATTAGCCCT

*BnaA.AP3.a* (91) .................G.....G.................G................................................

*BnaC.AP3.a* (91) .................A.....A.................T................................................

D5 (91) .................A.....A.................T................................................

*BolC.AP3.a* (91) .................A.....A.................G................................................

*BraA.AP3.a* (181) AACACCACAACAAAGGAGATCA--------------------------------------------------------------------

*BnaA.AP3.a* (181) ......................--------------------------------------------------------------------

*BnaA.AP3.a* (181) ......................--------------------------------------------------------------------

D5 (181) ......................AACTTTTTGTATTCGAAAACGATGTCCCTTTATGGGAGGAGATTGAATTAAAAAAAAAAAAAAAAAAA

*BolC.AP3.a*  (181) ......................--------------------------------------------------------------------

*BraA.AP3.a* (203) ------------------TAGATCTGTACCAAACCGTTTCTGATGTTGATGTTTGGAGCGCACACTATGAGAGAATGCAAGAAACCAAGA

*BnaA.AP3.a* (203) ------------------......................C....................A............................

*BnaC.AP3.a* (203) ------------------......................T....................T............................

D5 (271) AAAAAAAAAAGGAGATCA......................T....................T............................

*BolC.AP3.a* (203) ------------------......................T....................T............................

*BraA.AP3.a* (275) GGAAGCTGTTGGAGACAAATAGAAAGCTTCGGACTCAGATTAAGCAGAGGCTAGGTGAGTGTTTGGACGAACTTGATATTCAGGAGCTGC

*BnaA.AP3.a* (275) ............................C...........................................................T.

*BnaC.AP3.a* (275) ............................C...........................................................G.

D5 (361) ............................C...........................................................G.

*BolC.AP3.a* (275) ............................C...........................................................G.

*BraA.AP3.a* (365) GTAGTCTTGAGGAAGAAATGGAAAACACTTTCAAACTCGTGCGTGAGCGCAAGTTTAAATCCCTTGGAAATCAGATCGAAACCACCAAGA

*BnaA.AP3.a* (365) ........................................T..........................G......................

*BnaC.AP3.a* (365) ........................................T..........................G......................

D5 (451) ........................................T..........................G......................

*BolC.AP3.a* (365) ........................................T..........................G......................

*BraA.AP3.a* (455) AAAAGAACAAAAGTCAACAAGACATACAAAAGAATCTCATACATGAGCTGGAGCTAAGGGCAGAAGATCCTCACTATGGCCTAGTAGACA

*BnaA.AP3.a*  (455) ..........................................................A....................C..A..A....

*BnaC.AP3.a* (455) ..........................................................G....................A..T..G....

D5 (541) ..........................................................G....................A..T..G....

*BolC.AP3.a* (455) ..........................................................G....................A..T..A....

*BraA.AP3.a* (545) ATGGAGGCGACTACGATTCGGTTCTTGGATATCAAATCGAAGGATCACGTGCTTACGCTCTTCGTTACCATCAGAACCATCATCACCATT

*BnaA.AP3.a* (545) ...................G........A......................................C...........T..........

*BnaC.AP3.a* (545) ...................T........G......................................T...........G..........

D5 (631) ...................T........G......................................T...........G..........

*BolC.AP3.a*  (545) ...................T........G......................................T...........T..........

*BraA.AP3.a* (635) ACCCCAACCATGCCCTTCATGCACCATCTGCCTCTGACATCATTACCTTCCACCTTCTTGAATAA

*BnaA.AP3.a* (635) ...........A..............................................T....A.

*BnaC.AP3.a* (635) ...........G..............................................T....A.

D5 (721) ...........G..............................................T....A.

*BolC.AP3.a* (635) ...........G..............................................C....G.

**Figure S6. Nucleotide alignment of *B.AP3.a* among *B.rapa* and *B.oleracea* and *B.napus*.**

Note：*BraA.AP3.a*: *B.rapa*; *BolC.AP3.a*: *B.oleracea*; *BnaA.AP3.a* and *BnaC.AP3.a*: *B. napus*; D5: *BnaC.AP3.a* of Apt, AMSb and AMSa with an 86-bp foreign inserted sequence.
