# Supplementary material for: Functional Analysis of the Two Brassica AP3 Genes Involved in Apetalous and Stamen Carpelloid Phenotypes
Source: PLoS One. 2011 Jun 30;6(6):e20930. doi: 10.1371/journal.pone.0020930 (PMC3128040; doi:10.1371/journal.pone.0020930)
Supplement: Figure S7 — Flowers and AP3 genes distribution of F1 hybrids between AMS and HGMSII. (A) Flowers and petals of F1 hybrids between AMS and HGMSII. Fertile plant and SC male sterile plant in HGMSIIa×AMSb were named HAIIb and HAIIa, in AMSa×HGMSIIb were named AHIIb and AHIIa; (B) DNA amplification from HAIIb, HAIIa, AHIIb and AHIIa using 24-F and 24-R primers. Fertile plants AHIIb contained a B.AP3.a gene from male parent HGMSIIb, and their petals and stamens developed normally; (C) DNA amplification from HAb and HAa using AP3-F and AP3-R primers. Fertile plants HAIIb had a BnaA.AP3.b gene from AMSb, their stamens developed normally while petals showed sepaloid identity. (DOC) [file pone.0020930.s007.doc]

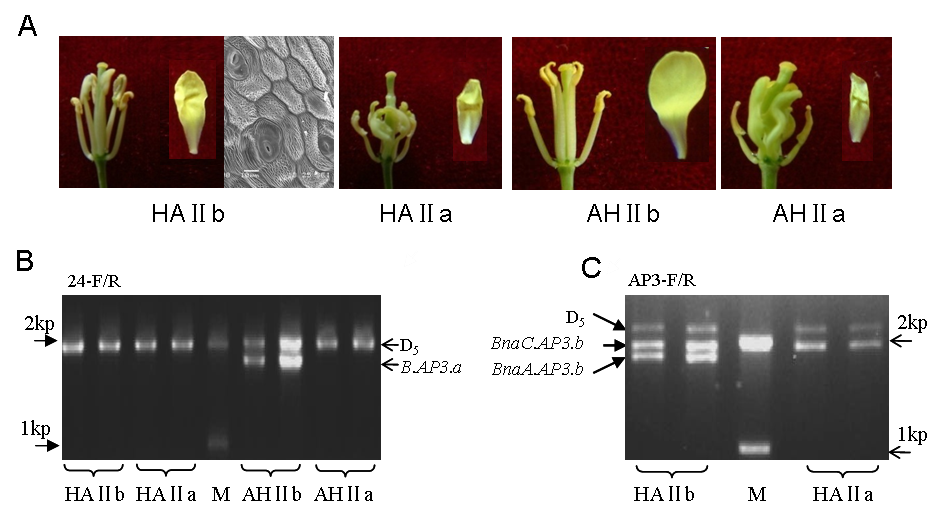


**Figure S7. Flowers and *AP3* genes distribution of F_1_ hybrids between AMS and HGMSII.**

(A) Flowers and petals of F_1_ hybrids between AMS and HGMSII. Fertile plant and SC male sterile plant in HGMSIIa×AMSb were named HAIIb and HAIIa, in AMSa×HGMSIIb were named AHIIb and AHIIa;

(B) DNA amplification from HAIIb, HAIIa, AHIIb and AHIIa using 24-F and 24-R primers. Fertile plants AHIIb contained a *B.AP3.a* gene from male parent HGMSIIb, and their petals and stamens developed normally;

(C) DNA amplification from HAb and HAa using AP3-F and AP3-R primers. Fertile plants HAIIb had a *BnaA.AP3.b* gene from AMSb, their stamens developed normally while petals showed sepaloid identity.
