# Supplementary material for: Functional Analysis of the Two Brassica AP3 Genes Involved in Apetalous and Stamen Carpelloid Phenotypes
Source: PLoS One. 2011 Jun 30;6(6):e20930. doi: 10.1371/journal.pone.0020930 (PMC3128040; doi:10.1371/journal.pone.0020930)
Supplement: Table S1 — Segregation of SC sterile lines HGMS and AMS by inter-sibling and self-crossings. (DOC) [file pone.0020930.s008.doc]

| **Table S1 Segregation of SC sterile lines HGMS and AMS by inter-sibling and self-crossings** | | | | | | | | | | | | |
| --- | --- | --- | --- | --- | --- | --- | --- | --- | --- | --- | --- | --- |
| **Type** | **Year** | **Line** | **Sterile plant × Fertile plant** | | | | | **Fertile plant by self-crossing** | | | | |
|  |  |  | **Fertile**  **plant** | **Sterile plant** | **Isolation rate** | **Expected**  **value** | **χ^2^** | **Fertile plants** | **Sterile plants** | **Isolation rate** | **Expected**  **value** | **χ^2^** |
| **HGMS** | **2002** | **01v635** | **67** | **55** | **1.2:1** | **1:1** | **1.180** | **34** | **14** | **2.4:1** | **3:1** | **0.444** |
|  | **2003** | **02v750** | **59** | **60** | **1.0:1** | **1:1** | **0.008** | **58** | **18** | **3.2:1** | **3:1** | **0.070** |
|  |  | **02v762** | **39** | **38** | **1.0:1** | **1:1** | **0.013** | **47** | **14** | **3.4:1** | **3:1** | **0.137** |
|  | **2004** | **03v645** | **419** | **399** | **1.05:1** | **1:1** | **0.441** | **320** | **115** | **2.78:1** | **3:1** | **0.405** |
| **AMS** | **2000** | **98Hx752** | **46** | **43** | **1.07:1** | **1:1** | **0.045** | **21** | **7** | **3.0:1** | **3:1** | **0** |
|  |  | **98Hx754** | **184** | **175** | **1.05:1** | **1:1** | **0.178** | **193** | **71** | **2.7:1** | **3:1** | **0.409** |
|  | **2001** | **99Hx799** | **830** | **770** | **1.08:1** | **1:1** | **2.176** | **697** | **210** | **3.3:1** | **3:1** | **1.553** |
|  | **2002** | **20E_2_N_2_** | **696** | **662** | **1.05:1** | **1:1** | **0.802** | **/** | **/** | **/** | **/** | **/** |
| Note: χ^2^_0.05，1_ =3.84; Sterile plant means the SC male sterile plant. | | | | | | | | | | | | |
